# Development of Amylose- and β-Cyclodextrin-Based Chiral Fluorescent Sensors Bearing Terthienyl Pendants

**DOI:** 10.3390/molecules21111518

**Published:** 2016-11-11

**Authors:** Tomoyuki Ikai, Changsik Yun, Yutaka Kojima, Daisuke Suzuki, Katsuhiro Maeda, Shigeyoshi Kanoh

**Affiliations:** Graduate School of Natural Science and Technology, Kanazawa University, Kakuma-machi, Kanazawa 920-1192, Japan; robiny87@gmail.com (C.Y.); y_kojima_1991@yahoo.co.jp (Y.K.); dsuke3heat@gmail.com (D.S.); maeda@se.kanazawa-u.ac.jp (K.M.); kanoh@se.kanazawa-u.ac.jp (S.K.)

**Keywords:** carbohydrates, chirality, fluorescent sensor, helix, higher-order structure, polysaccharides

## Abstract

Phenylcarbamate derivatives of amylose and β-cyclodextrin show excellent chiral recognition when used as chiral stationary phases (CSPs) for high-performance liquid chromatography. To open up new possibilities of carbohydrate-based materials, we developed chiral fluorescent sensors based on amylose and β-cyclodextrin (Am-**1b** and CyD-**1b**, respectively) by attaching fluorescent π-conjugated units on their side chains. Their recognition abilities toward chiral analytes containing a nitrophenyl unit were evaluated by measuring the enantioselective fluorescence quenching behavior. Both sensors showed the same degree of enantioselective fluorescence response for various aromatic nitro compounds. However, in some cases, their enantioselectivities were different depending on the analytes. The difference in the chiral recognition abilities between Am-**1b** and CyD-**1b** seems to be based on the structural difference of their inherent backbones, that is, the one-handed helical structure and cyclic structure, respectively. The study on the resolution ability of the Am-**1b**-based CSP revealed that the terthienyl-based pendant of Am-**1b** provides not only a fluorescent functionality but also a different chiral recognition site from that of amylose tris(phenylcarbamate).

## 1. Introduction

**Figure 1:**
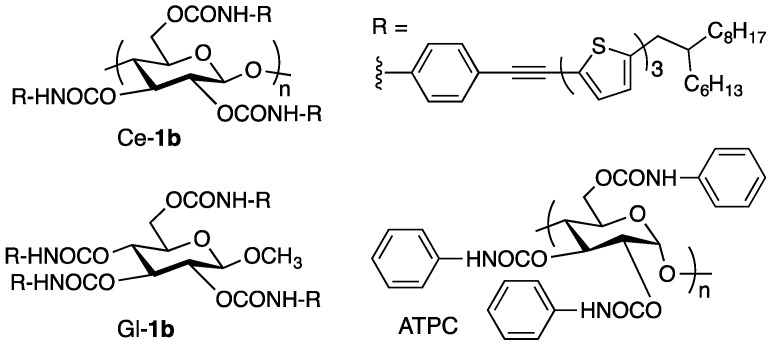
Structures of previously reported phenylcarbamate derivatives of polysaccharides (Ce-**1b** and ATPC) and glucose (Gl-**1b**).

Biologically interesting chiral molecules are ubiquitous in the fields of drugs, agrochemicals, food additives and fragrances. It has been widely recognized that the physiological properties of two enantiomers are often different owing to the sophisticated chiral discrimination ability of enzymes and receptors in the body [1,2,3,4]. Thus, to eliminate the incorrect use of hazardous isomers that may have undesired properties, chiral discrimination has been viewed as an important issue, particularly in the pharmaceutical industries. Polysaccharides, such as amylose and cellulose, are renewable and abundant resources that are optically active. Although natural polysaccharides hardly show marked chiral discrimination ability [5,6], their appropriately modified derivatives (e.g., phenylcarbamate derivatives) are known to show excellent chiral recognition for a wide variety of racemic compounds when applied to chiral stationary phases (CSPs) for high-performance liquid chromatography (HPLC) [7,8,9,10]. Today, polysaccharide-based CSPs are widely used around the world, not only for the analysis of enantiomeric compositions but also to obtain optically pure enantiomers in the bulk. Although great success has been achieved in the application of polysaccharide derivatives in CSP materials, their application in other fields of chiral materials has been highly restricted. We have reported functionalized polysaccharide derivatives bearing reactive prochiral units or catalytically active sites as pendant groups with the aim of developing chiral auxiliaries or asymmetric catalysts, respectively, for asymmetric synthesis [11,12]. In addition, a cellulose-based chiral fluorescent sensor (Ce-**1b** in Figure 1) containing a terthienyl moiety as a fluorescent signaling unit has also been developed and was found to show apparent enantioselectivity for various types of chiral compounds containing either central or axial chirality [13]. The significant contribution of the helical chirality of Ce-**1b** to the chiral fluorescence sensing was demonstrated by the fact that the corresponding monosaccharide-based model compound (Gl-**1b** in Figure 1) showed almost no enantioselectivity.

**Scheme 1:**
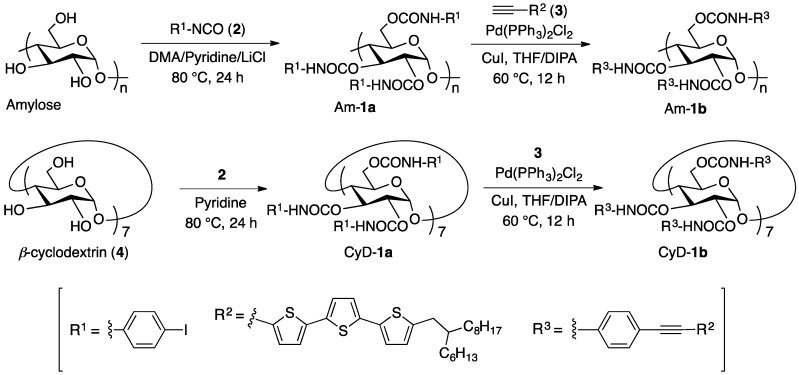
Synthesis of amylose (Am-**1b**) and β-cyclodextrin (CyD-**1b**) derivatives bearing terthienyl pendants.

In the present study, we developed a novel amylose-based chiral fluorescent sensor (Am-**1b** in Scheme 1) bearing terthienyl-based π-conjugated pendants as fluorescent signaling units. We compared the enantioselective fluorescent responses of Am-**1b** toward chiral aromatic nitro compounds with that of the corresponding β-cyclodextrin derivative (CyD-**1b** in Scheme 1), which has the same repeating unit as Am-**1b**. Although a large number of chiral fluorescence sensors, including β-cyclodextrin-based sensors, have been developed so far [14,15,16], to the best of our knowledge, there have been no reports of the amylose-based chiral fluorescence sensors except for the seminal example of chiral sensing using amylose-functionalized graphene [17].

## 2. Results and Discussion

The amylose derivative (Am-**1b**) bearing fluorescent terthienyl-based π-conjugated pendants at the 2-, 3- and 6-positions of the glucose unit was synthesized according to our previously reported procedure (Scheme 1) [13]. Am-**1b** showed good solubility, not only in common organic solvents, such as tetrahydrofuran (THF) and chloroform but also in mixtures of these solvents with 90 vol % hexane. Hexane is typically a poor solvent for the previously reported amylose phenylcarbamates used as CSPs for HPLC. The ample solubility of Am-**1b** is probably owing to the branched alkyl chains at the terminal thiophene ring of the π-conjugated pendants. We also prepared the corresponding phenylcarbamate derivative of β-cyclodextrin (CyD-**1b**) bearing the same terthienyl pendants using a similar procedure.

**Figure 2:**
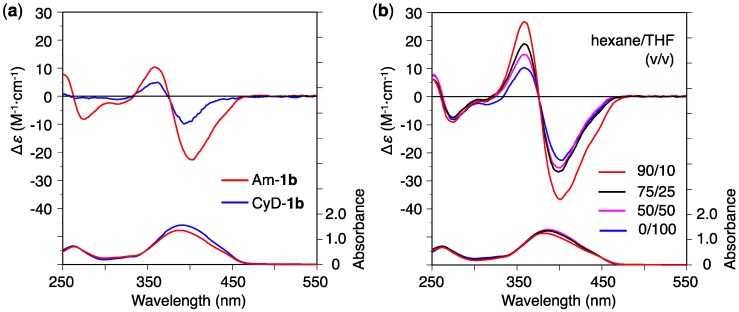
(**a**) CD and absorption spectra of Am-**1b** and CyD-**1b** in THF at 25 °C; (**b**) CD and absorption spectra of Am-**1b** in hexane/THF (hexane: 0–90 vol %) at 25 °C. [Glucose unit] = 1.0 × 10^−5^ M.

Figure 2a shows the circular dichroism (CD) and absorption spectra of Am-**1b** and CyD-**1b** in THF. An apparent split-type CD absorption was observed in the absorption region of the terthienyl pendant of Am-**1b** (330–460 nm). As the previously reported glucose derivative Gl-**1b** showed a very weak CD absorption in this region [13], the CD absorption of Am-**1b** is considered to not be derived from the central chirality of the glucose ring. An asymmetric arrangement is most likely induced in the pendants of Am-**1b** owing to the helical structure of the amylose phenylcarbamate framework, which is known to have a left-handed 4/3 helical conformation [18,19]. The chiroptical properties of Am-**1b** were also measured in hexane/THF mixtures (hexane: 50 vol %–90 vol %) (Figure 2b), which is a solvent used in the following section for chiral fluorescent sensing. The CD intensities of Am-**1b** increased with increasing hexane content. The hypochromic effect was also observed in its absorption spectrum that was measured in hexane/THF (90/10, *v*/*v*). Figure S1a shows the dynamic light scattering (DLS) analysis of Am-**1b** in THF, from which the hydrodynamic diameter (*D*_h_) of Am-**1b** in THF was estimated to be approximately 53 nm. This value was almost comparable to that measured in hexane/THF (90/10, *v*/*v*) (approximately 51 nm) (Figure S1b). These results indicate that the spectral change induced by the addition of high amounts of hexane was mainly caused by a certain alteration in the arrangement of the π-conjugated pendants through intramolecular interactions. Importantly, the CD spectral pattern of Am-**1b** was found to be almost a mirror image of the corresponding cellulose phenylcarbamate Ce-**1b** (Figure S2). This suggests that the π-conjugated pendants of Am-**1b** and Ce-**1b** are arranged in opposite chiral environments to each other, probably reflecting their higher-order structures. CyD-**1b** also showed a similar split-type CD absorption in the terthienyl chromophore region, although its CD intensity and pattern were different from those of Am-**1b**, particularly in the absorption region below 300 nm. In a hexane/THF mixture (90/10, *v*/*v*), the CD intensity of CyD-**1b** was increased and its pattern showed a close resemblance to that of Am-**1b** over the entire absorption region (Figure S3). This indicated that the π-conjugated pendants of CyD-**1b** were arranged in a similar manner to those of Am-**1b** only when the solution contained a high content of hexane (~90 vol %).

**Figure 3:**
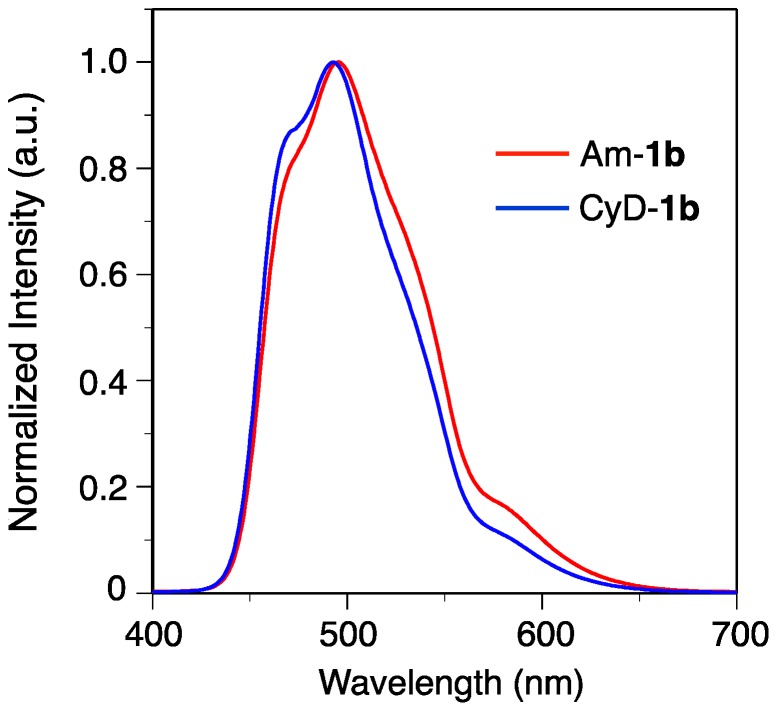
Fluorescence spectra of Am-**1b** (λ_ex_ = 364 nm) and CyD-**1b** (λ_ex_ = 362 nm) in THF at room temperature. [Glucose unit] = 1.0 × 10^−5^ M.

The fluorescence spectra of Am-**1b** and CyD-**1b** measured in THF are shown in Figure 3. As depicted in the photographs of their solutions that were irradiated at 365 nm (Figure S4), apparent greenish emissions derived from their π-conjugated pendants were clearly observed in both cases. Am-**1b** and CyD-**1b** exhibited fluorescence quantum yields of 5% and 6%, respectively, in THF and 2% and 3%, respectively, in hexane/THF (90/10, *v*/*v*), which are low but sufficient for use as fluorescent sensor materials. As was the case for Ce-**1b** [13], the fluorescence intensities of the present fluorophores were diminished when nitrobenzene (**5**) was added to the solutions (Figure S4). This was most likely as a result of photoinduced electron transfer from the fluorescent pendant residues to **5**. This fluorescence quenching behavior prominently appeared in a THF solution containing 90 vol % hexane as a nonpolar solvent (Figures S4b,d). This result indicated that THF molecules compete with **5** in the interaction with Am-**1b** and that the hydrogen-bonding and/or the dipole–dipole interactions between these fluorophores and **5** play an important role in the fluorescence quenching.

**Figure 4:**
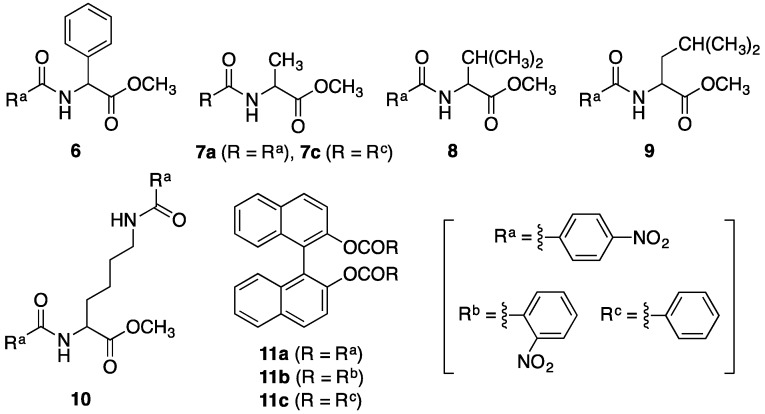
Structures of chiral aromatic nitro compounds and their analogs (**6**–**11**).

**Figure 5:**
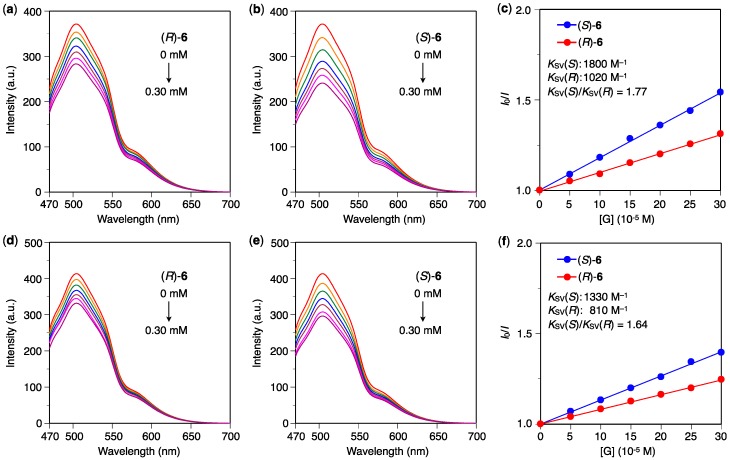
Fluorescence spectra of Am-**1b** (**a**,**b**) and CyD-**1b** (**d**,**e**) upon the addition of various amounts of (*R*)-**6** (**a**,**d**) and (*S*)-**6** (**b**,**e**) in hexane/THF (90/10, *v*/*v*) at room temperature. [Glucose unit] = 1.0 × 10^−5^ M, [**6**] = 0–0.30 mM, λ_ex_ = 450 nm; (**c**,**f**) Stern–Volmer plots for the fluorescence quenching of Am-**1b** (**c**) and CyD-**1b** (**f**) by (*R*)-**6** (red) and (*S*)-**6** (blue).

Next, we investigated the enantioselective fluorescent responses of Am-**1b** and CyD-**1b** toward chiral aromatic nitro compounds (**6**–**11** in Figure 4). When the fluorescence spectra of Am-**1b** were measured in the presence of (*R*)-**6** or (*S*)-**6** in a hexane/THF (90/10, *v*/*v*) mixture (Figure 5a,b), the fluorescence intensities gradually decreased with increasing concentration of either enantiomer. Figure 5c shows the Stern–Volmer plots for the fluorescence quenching of Am-**1b** by the (*R*)- and (*S*)-isomers of **6**. We found that (*S*)-**6** quenched the emission of Am-**1b** more efficiently than (*R*)-**6** and that the fluorescence quenching obeys the Stern–Volmer equation (Equation (1)) in the concentration range from 0 to 0.30 mM of **6**, resulting in straight lines for both isomers:

*I*_0_/*I* = *K*_SV_[Q] + 1 (1)

In this equation, *I* and *I*_0_ are the fluorescence intensities of Am-**1b** at 450 nm with and without a guest quencher, respectively, *K*_SV_ is the Stern–Volmer constant, and [Q] is the concentration of the guest. From these plots, the *K*_SV_ values for (*S*)-**6** and (*R*)-**6** were determined to be 1800 and 1020 M^−1^, respectively, with a ratio of 1.77.

**Figure 6:**
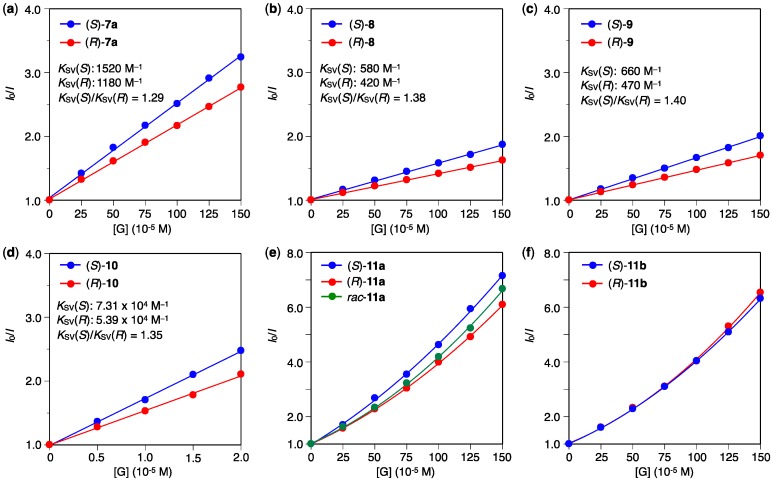
Stern–Volmer plots for the fluorescence quenching of Am-**1b** (λ_ex_ = 450 nm) by the (*R*)- (red) and (*S*)- (blue) isomers of **7a** (**a**); **8** (**b**); **9** (**c**); **10** (**d**); **11a** (**e**) and **11b** (**f**) in hexane/THF (90/10, *v*/*v*) at room temperature. The result using *rac*-**11a** as a quencher is also shown in (**e**). [Glucose unit] = 1.0 × 10^−5^ M.

Am-**1b** showed distinct enantioselective fluorescence responses toward all of the tested aromatic nitro compounds except for **11b** (Figure 6). The upward curvatures observed in the Stern–Volmer plots using **11** as quenchers indicate that the fluorescence quenching of Am-**1b** by **11** occurs by both dynamic and static quenching processes (Figure 6e,f) [20]. We also confirmed that an intermediate quenching between the results obtained using (*R*)-**11a** and (*S*)-**11a** was observed when *rac*-**11a** was used as a quencher (Figure 6e). In all cases except for **11b**, the fluorescence emission from Am-**1b** was more strongly quenched by each (*S*)-isomer. This means that the intermolecular interactions between Am-**1b** and the (*S*)-isomers are more effective than those with the corresponding antipodes. Interestingly, the enantioselectivities observed for Am-**1b** were opposite to those observed for the corresponding cellulose-based sensor (Ce-**1b**), in which the (*R*)-isomers induced more noticeable quenching than their antipodes (Figure S5) [13]. As described in the CD spectral analysis, the π-conjugated pendants in Am-**1b** seem to be arranged in opposite chiral environments to those of Ce-**1b**, which may result in different enantioselectivities in the fluorescent sensing.

**Figure 7:**
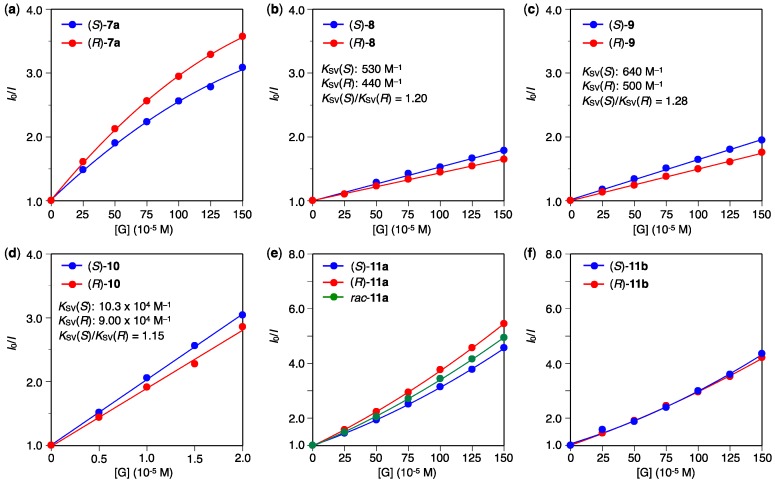
Stern–Volmer plots for the fluorescence quenching of CyD-**1b** (λ_ex_ = 450 nm) by the (*R*)- (red) and (*S*)- (blue) isomers of **7a** (**a**); **8** (**b**); **9** (**c**); **10** (**d**); **11a** (**e**) and **11b** (**f**) in hexane/THF (90/10, *v*/*v*) at room temperature. The result using *rac*-**11a** as a quencher is also shown in (**e**). [Glucose unit] = 1.0 × 10^−5^ M.

CyD-**1b** also exhibited an enantioselective fluorescence response to aromatic nitro compounds (Figure 5d–f and Figure 7). The Stern–Volmer plots obtained using **7a** as quenchers show non-linear relationships, suggesting that some of the π-conjugated pendants were inaccessible or only slightly accessible to the guest quenchers (Figure 7a) [20]. The chiral recognition ability of CyD-**1b** was found to be almost comparable to that of Am-**1b**, although the enantioselectivities against **7a** and **11a** were opposite to each other. As both fluorophores were composed of the same α-d-glucose-based repeating unit, the characteristic chiral sensing abilities observed for Am-**1b** and CyD-**1b** are most likely present as a result of the formation of a one-handed helical structure and a cyclic structure, respectively, rather than the contribution from the monomeric glucose unit itself. This reasoning is also supported by the following results that have been reported previously. (1) A distinct chiral recognition was hardly observed using Gl-**1b** as a sensor [13]; (2) Amylose phenylcarbamates showed different resolution abilities to the corresponding β-cyclodextrin derivatives when used as CSPs for HPLC [21]. It was also found that *rac*-**7c** and *rac*-**11c** without a nitro group did not quench the emission of Am-**1b** and CyD-**1b** at all, probably due to a mismatch in the lowest unoccupied molecular orbital levels of the fluorophores and the guests (Figure S6).

The influence of the fluorescent terthienyl-based pendant on the chiral recognition was investigated by comparison of the chiral recognition abilities of Am-**1b** and the previously reported amylose tris(phenylcarbamate) (ATPC in Figure 1), which contains non-substituted phenylcarbamate pendants [22]. Because the enantioselectivity of ATPC cannot be evaluated as a chiral fluorescence sensor due to the lack of fluorescence, their chiral recognition abilities were compared on the basis of their resolution abilities when used as CSPs for HPLC. The Am-**1b**- and ATPC-based CSPs were prepared by coating the corresponding polymers on macroporous silica gel. Their resolution abilities were evaluated using the aromatic nitro compounds and their analogs (*rac*-**6**–**11**). Because Am-**1b** had a high affinity for hexane and was soluble in a hexane/alcohol mixture, which is a typical eluent for chiral HPLC, ethanol was used as the eluent. The CyD-**1b**-based CSP was slightly soluble in ethanol; therefore, the evaluation of its resolution ability was impossible. The results of the resolution of *rac*-**11a**–**c** are summarized in Table 1, and the chromatogram of *rac*-**11a** partially resolved on Am-**1b** is shown in Figure S7 [23]. The enantiomers were eluted at retention times of *t*_1_ and *t*_2_, and the hold-up time (*t*_0_) was estimated to be 18.7 min. Therefore, the retention factors, *k*_1_ [=(*t*_1_ − *t*_0_)/*t*_0_] and *k*_2_ [=(*t*_2_ − *t*_0_)/*t*_0_], were calculated to be 1.15 and 1.35, respectively, resulting in a separation factor α (=*k*_2_/*k*_1_) of 1.17.

The Am-**1b**-based CSP displayed a clear resolving ability only toward **11a** among three racemates **11a**–**11c**, whereas ATPC resolved only **11b**. The different resolution abilities of these CSPs imply that the terthienyl-based pendant of Am-**1b** provides not only a fluorescent functionality but also a totally different recognition site from that of the original ATPC. In the resolution of *rac*-**11a** on the Am-**1b**-based CSP, the (*R*)-isomer was eluted first followed by the (*S*)-isomer, meaning that Am-**1b** interacts more favorably with the (*S*)-isomer. Combined with the finding that the Am-**1b**-based CSP could not resolve **11b**, the enantioselectivity of the Am-**1b**-based CSP is in agreement with the fluorescence sensing results. The fact that Am-**1b** did not show clear recognition ability toward **11c** suggests that the nitro group of **11a** probably acts as a valuable interaction site with Am-**1b**.

## 3. Materials and Methods

### 3.1. General Information

Anhydrous solvents, such as THF, dichloromethane, pyridine and *N*,*N*-dimethylacetamide (DMA), as well as lithium chloride were purchased from Kanto Kagaku (Tokyo, Japan). Amylose (DP~300) was kindly supplied by Daicel Chemical Industries (Tokyo, Japan). 4-Iodophenyl isocyanate (**2**) and copper (I) iodide (CuI) were from Sigma-Aldrich (St. Louis, MO, USA). β-Cyclodextrin (**4**), *trans*-dichlorobis(triphenylphosphine)palladium(II) (Pd(PPh_3_)_2_Cl_2_), 1-ethyl-3-(3-dimethylaminopropyl)carbodiimide hydrochloride (EDC-HCl) and *N*,*N*-dimethyl-4-aminopyridine (DMAP), diisopropylamine (DIPA) and **5** were purchased from Wako Pure Chemical Industries (Osaka, Japan). Triethylamine was obtained from Kishida (Osaka, Japan). All starting materials for the synthesis of aromatic nitro compounds and their analogues (**6**–**11**) were purchased from Wako Pure Chemical Industries, Tokyo Kasei Kogyo (TCI) (Tokyo, Japan) and Nacalai (Kyoto, Japan). Aromatic nitro compounds and their analogues (**7**–**9**, **11a** and **11b**) were prepared according to literature procedures [13]. The porous spherical silica gel with a mean particle size of 5 μm and a mean pore diameter of 100 nm (Daiso gel SP-1000-5) was kindly supplied from OSAKA SODA (Osaka, Japan). Chiralpak IA (25 cm × 0.46 cm (i.d.)) was purchased from Daicel (Tokyo, Japan). ATPC [22] and a terthiophene compound bearing an ethynyl group (**3**) [13] were prepared according to literature procedures.

### 3.2. Synthesis

Aromatic nitro compounds **6**, **10** and **11c** were synthesized thorough common condensation reactions using the corresponding amine or alcohol compounds as starting materials.

*(−)-N-(4-Nitrobenzoyl)-d-phenylglycine methyl ester* ((*R*)-**6**). d-Phenylglycine methyl ester hydrochloride (0.40 g, 2.0 mmol) and triethylamine (0.84 mL, 6.0 mmol) were dissolved in anhydrous THF (5 mL) and the solution was cooled to 0 °C. To this solution was added 4-nitrobenzoyl chloride (0.37 g, 2.0 mmol) dissolved in THF (5 mL) and the mixture was stirred at r.t. for 2 h. The mixture was diluted with THF, washed with brine and then dried over Na_2_SO_4_. The solvent was removed under reduced pressure, followed by recrystallization from a hexane–ethyl acetate mixture to give the desired product as a white solid (0.48 g, 76% yield). m.p.: 189.1–189.4 °C. [α]${}_{D}^{25}$ −6.8 (*c* 1.0, DMSO). ^1^H-NMR (500 MHz, DMSO-*d*_6_, r.t.): δ 9.58 (d, *J* = 6.9 Hz, 1H, NH), 8.32 (d, *J* = 8.6 Hz, 2H, ArH), 8.14 (d, *J* = 9.0 Hz, 2H, ArH), 7.49 (d, *J* = 7.0 Hz, 2H, ArH), 7.44–7.35 (m, 3H, ArH), 5.69 (d, *J* = 6.9 Hz, 1H, CH), 3.67 (s, 3H, OCH_3_). ^13^C-NMR (125 MHz, DMSO-*d*_6_, r.t.): δ 170.83, 165.07, 149.25, 139.13, 135.77, 129.35, 128.66, 128.41, 128.33, 123.47, 57.14, 52.42. IR (KBr, cm^−1^): 3322 (N-H), 1746 (C=O). Calcd for C_16_H_14_N_2_O_5_: C, 61.14; H, 4.49; N, 8.91. Found: C, 61.03; H, 4.38; N, 8.93.

*(+)-N-(4-Nitrobenzoyl)-l-phenylglycine methyl ester* ((*S*)-**6**). The title compound was prepared from l-phenylglycine methyl ester hydrochloride in the same way as (*R*)-**6** and obtained in 56% yield as a white solid. m.p.: 189.0–189.3 °C. [α]${}_{D}^{25}$ +7.0 (*c* 1.0, DMSO). ^1^H-NMR (500 MHz, DMSO-*d*_6_, r.t.): δ 9.58 (d, *J* = 6.9 Hz, 1H, NH), 8.32 (d, *J* = 9.2 Hz, 2H, ArH), 8.14 (d, *J* = 9.2 Hz, 2H, ArH), 7.49 (d, *J* = 6.5 Hz, 2H, ArH), 7.44–7.35 (m, 3H, ArH), 5.69 (d, *J* = 6.9 Hz, 1H, CH), 3.67 (s, 3H, OCH_3_). ^13^C-NMR (125 MHz, DMSO-*d*_6_, r.t.): δ 170.81, 165.05, 149.23, 139.11, 135.75, 129.33, 128.65, 128.40, 128.32, 123.46, 57.13, 52.41. IR (KBr, cm^−1^): 3321 (N-H), 1746 (C=O). Calcd for C_16_H_14_N_2_O_5_: C, 61.14; H, 4.49; N, 8.91. Found: C, 61.06; H, 4.47; N, 8.89.

*(+)-N-(4-Nitrobenzoyl)-d-lysine methyl ester* ((*R*)-**10**). The title compound was prepared from d-lysine methyl ester hydrochloride in the same way as (*R*)-**6** and obtained in 20% yield as a white solid. Mp: 201.6–201.9 °C. [α]${}_{D}^{25}$ + 0.9 (*c* 1.0, DMSO). ^1^H-NMR (500 MHz, DMSO-*d*_6_, 80 °C): δ 8.83 (d, *J* = 6.9 Hz, 1H, NH), 8.55 (s, 1H, NH), 8.27 (d, *J* = 8.5 Hz, 2H, ArH), 8.25 (d, *J* = 8.5 Hz, 2H, ArH), 8.08 (d, *J* = 9 Hz, 2H, ArH), 8.03 (d, *J* = 8.5 Hz, 2H, ArH), 4.54–4.48 (m, 1H, CH), 3.67 (s, 3H, OCH_3_), 3.32 (q, *J* = 6.5 Hz, 2H, CH_2_), 1.96–1.81 (m, 2H, CH_2_), 1.68–1.56 (m, 2H, CH_2_), 1.54–1.42 (m, 2H, CH_2_). ^13^C-NMR (125 MHz, DMSO-*d*_6_, r.t.): δ 172.46, 165.06, 164.46, 149.13, 148.86, 140.23, 139.24, 128.98, 128.62, 123.50, 123.45, 52.77, 51.99, 39.10, 30.03, 28.39, 23.10. IR (KBr, cm^−1^): 3305 (N-H), 1731 (C=O). Calcd for C_21_H_22_N_4_O_8_·0.3H_2_O: C, 54.38; H, 4.91; N, 12.08. Found: C, 54.22; H, 4.76; N, 11.97.

*(–)-N-(4-Nitrobenzoyl)-l-lysine methyl ester* ((*S*)-**10**). The title compound was prepared from l-lysine methyl ester hydrochloride in the same way as (*R*)-**6** and obtained in 21% yield as a white solid. m.p.: 201.5–201.8 °C. [α]${}_{D}^{25}$ −1.2 (*c* 1.0, DMSO). ^1^H-NMR (500 MHz, DMSO-*d*_6_, 80 °C): δ 8.83 (d, *J* = 7.4 Hz, 1H, NH), 8.55 (s, 1H, NH), 8.27 (d, *J* = 9.5 Hz, 2H, ArH), 8.25 (d, *J* = 9 Hz, 2H, ArH), 8.08 (d, *J* = 9 Hz, 2H, ArH), 8.03 (d, *J* = 8.5 Hz, 2H, ArH), 4.54–4.48 (m, 1H, CH), 3.67 (s, 3H, OCH_3_), 3.32 (q, *J* = 6.5 Hz, 2H, CH_2_), 1.96–1.81 (m, 2H, CH_2_), 1.68–1.56 (m, 2H, CH_2_), 1.53–1.41 (m, 2H, CH_2_). ^13^C-NMR (125 MHz, DMSO-*d*_6_, r.t.): δ 172.48, 165.09, 164.49, 149.15, 148.88, 140.24, 139.26, 129.00, 128.64, 123.52, 123.47, 52.79, 52.01, 39.17, 30.04, 28.41, 23.11. IR (KBr, cm^−1^): 3305 (N-H), 1730 (C=O). Calcd for C_21_H_22_N_4_O_8_·0.2H_2_O: C, 54.59; H, 4.89; N, 12.13. Found: C, 54.53; H, 4.75; N, 12.05.

*(R)-(+)-1,1′-Binaphthyl-2,2′-diyl bis(2-nitrobenzoate)* ((*R*)-**11c**). (*R*)-1,1′-Bi-2-naphthol (0.50 g, 1.8 mmol), 2-nitrobenzoic acid (0.87 g, 5.2 mmol), DMAP (0.64 g, 5.2 mmol) were dissolved in anhydrous dichloromethane (9 mL) and the solution was cooled to 0 °C. To this solution was added EDC-HCl (1.0 g, 5.2 mmol) and the mixture was stirred at r.t. for 12 h. The mixture was diluted with dichloromethane, washed with 1 N HCl aqueous solution and water and then dried over Na_2_SO_4_. The solvent was removed under reduced pressure and the crude product was purified by silica gel chromatography using hexane–dichloromethane (1/4, *v*/*v*) as the eluent to give the desired product as a pale yellow solid (0.81 g, 79% yield). m.p.: 169.4–169.7 °C. [α]${}_{D}^{25}$ + 9.3 (*c* 1.0, CHCl_3_). ^1^H-NMR (400 MHz, CDCl_3_, r.t.): δ 8.11 (d, *J* = 11.0 Hz, 2H, ArH), 7.98 (d, *J* = 10.5 Hz, 2H, ArH), 7.91 (dd, *J* = 10.0, 1.5 Hz, 2H, ArH), 7.77 (d, *J* = 10.5 Hz, 2H, ArH), 7.54–7.29 (m, 10H, ArH), 6.76 (dd, *J* = 9.0, 2.5 Hz, 2H, ArH). ^13^C-NMR (125 MHz, CDCl_3_, r.t.): δ 164.36, 146.75, 146.54, 133.65, 133.35, 131.92, 131.42, 130.23, 129.24, 128.23, 127.84, 127.29, 126.31, 126.21, 124.02, 123.50, 121.36. IR (KBr, cm^−1^): 1752 (C=O). Calcd for C_34_H_20_N_2_O_8_: C, 69.86; H, 3.45; N, 4.79. Found: C, 69.96; H, 3.51; N, 4.75.

*(S)-(−)-1,1′-Binaphthyl-2,2′-diyl bis(2-nitrobenzoate)* ((*S*)-**11c**). The title compound was prepared from (*S*)-1,1′-bi-2-naphthol in the same way as (*R*)-**11c** and obtained in 84% yield as a pale yellow solid. m.p.: 169.2–169.5 °C. [α]${}_{D}^{25}$ −9.4 (*c* 1.0, CHCl_3_). ^1^H-NMR (500 MHz, CDCl_3_, r.t.): δ 8.11 (d, *J* = 9.5 Hz, 2H, ArH), 7.98 (d, *J* = 8.0 Hz, 2H, ArH), 7.91 (d, *J* = 8.0 Hz, 2H, ArH), 7.77 (d, *J* = 8.5 Hz, 2H, ArH), 7.53–7.30 (m, 10H, ArH), 6.76 (d, *J* = 7.5 Hz, 2H, ArH). ^13^C-NMR (125 MHz, CDCl_3_, r.t.): δ 164.34, 146.75, 146.53, 133.63, 133.34, 131.91, 131.41, 130.23, 129.23, 128.23, 127.82, 127.28, 126.31, 126.20, 124.00, 123.49, 121.35. IR (KBr, cm^−1^): 1747 (C=O). Calcd for C_34_H_20_N_2_O_8_: C, 69.86; H, 3.45; N, 4.79. Found: C, 70.00; H, 3.48; N, 4.75.

*Synthesis of Am-***1a**: Amylose (0.40 g, 2.5 mmol) was first dissolved in a mixture of DMA (12 mL), lithium chloride (0.80 g) and pyridine (6.0 mL). Then, **2** (1.80 g, 7.35 mmol) was added, and the mixture was stirred for 24 h at 80 °C. After the reaction, the target amylose derivative Am-**1a** (1.10 g, 50%) was isolated as the methanol-insoluble fraction. ^1^H-NMR (400 MHz, pyridine-*d*_5_, 80 °C): δ 10.70–9.80 (br, 3H, NH), 7.90–7.00 (br, 12H, ArH), 6.00–4.10 (br, 7H, glucose protons). IR (KBr, cm^−1^): 3386 (N-H), 1718 (C=O). Calcd for C_27_H_22_I_3_N_3_O_8_·0.2H_2_O: C, 36.00; H, 2.51; N, 4.66. Found: C, 35.72; H, 2.60; N, 4.63.

*Synthesis of Am-***1b**: To a solution of Am-**1a** (0.10 g, 0.11 mmol) and **3** (0.25 g, 0.50 mmol) in degassed THF/DIPA (5/1, *v*/*v*) (3 mL) were added Pd(PPh_3_)_2_Cl_2_ (23 mg, 33 μmol) and CuI (19 mg, 0.10 mmol), and then the mixture was heated to 60 °C. After 12 h, the reaction mixture was diluted with dichloromethane and passed through Celite to remove the metal catalyst. The solution was concentrated and poured into a large amount of ethanol, and the resulting polymer was collected by centrifugation and washed with ethanol for 12 h and then with hexane for 48 h in a Soxhlet apparatus to remove any by-products. After the polymer was collected in THF, the solution was concentrated and poured into methanol. The precipitate was collected by centrifugation and dried in vacuo to give Am-**1b** as an brown solid (0.16 g, 72%). ^1^H-NMR (500 MHz, CDCl_3_, 55 °C): δ 10.0–9.0 (br, 3H, NH), 8.15–6.00 (br, 30H, ArH), 6.20–3.25 (br, 7H, glucose protons), 2.83 (br, 6H, CH_2_), 1.60 (br, 3H, CH), 1.65–1.05 (br, 72H, CH_2_), 0.95–0.70 (br, 18H, CH_3_). IR (KBr, cm^−1^): 3398 (N-H), 2199 (C≡C), 1719 (C=O). Calcd for C_117_H_139_N_3_O_8_S_9_·0.5H_2_O: C, 69.81; H, 7.01; N, 2.09. Found: C, 69.53; H, 6.86; N, 2.05.

*Synthesis of CyD-***1a**: The title compound was prepared from **4** in the same way as Am-**1a** and obtained in 52% yield as a pale white solid. ^1^H-NMR (500 MHz, DMSO-*d*_6_, 80 °C): δ 9.46 (br, 7H, NH), 9.33 (br, 7H, NH), 7.96 (br, 7H, NH), 7.53 (d, *J* = 8.5 Hz, 14H, ArH), 7.31 (d, *J* = 9.0 Hz, 14H, ArH), 7.27 (d, *J* = 8.5 Hz, 14H, ArH), 7.20 (d, *J* = 8.5 Hz, 14H, ArH), 7.01 (d, *J* = 8.5 Hz, 14H, ArH), 6.75 (d, *J* = 8.5 Hz, 14H, ArH), 5.48 (t, *J* = 9.5 Hz, 7H, CH), 5.33 (br, 7H, CH), 4.83 (d, *J* = 13.0 Hz, 7H, CH), 4.70–4.55 (m, 14H, CH), 4.30 (d, *J* = 9.0 Hz, 7H, CH), 4.05 (t, *J* = 9.5 Hz, 7H, CH). IR (KBr, cm^−1^): 3394 (N-H), 1738 (C=O).

*Synthesis of CyD-***1b**: The title compound was prepared from CyD-**1a** in the same way as Am-**1b** and obtained in 72% yield as a yellow solid. ^1^H-NMR (500 MHz, CDCl_3_, 55 °C): δ 8.00–6.35 (m, 231H, ArH, NH), 5.60 (br, 7H, CH), 5.31 (br, 7H, CH), 5.10 (br, 7H, CH), 4.85 (br, 7H, CH), 4.64 (br, 7H, CH), 4.49 (br, 7H, CH), 3.93 (br, 7H, CH), 2.75–2.55 (m, 42H, CH_2_), 1.61 (br, 21H, CH), 1.40–1.10 (br, 504H, CH_2_), 0.95–0.83 (br, 126H, CH_3_). IR (KBr, cm^−1^): 3394 (N-H), 2200 (C≡C), 1744 (C=O). Calcd for C_819_H_973_N_21_O_56_S_63_: C, 70.13; H, 6.99; N, 2.10. Found: C, 69.84; H, 7.04; N, 1.87.

### 3.3. Sample Preparation for Fluorescence Measurements

All solutions were prepared using volumetric syringes and flasks. The stock solutions of the fluorophores and quenchers were freshly prepared and used for each measurement. For the fluorescence quenching study, a fluorophore solution (10^−4^ M) in THF or hexane/THF was mixed with a THF solution of the quencher in a 5 mL volumetric flask and diluted with THF and hexane to the desired concentration. The resulting solutions were allowed to stand at 25 °C for 1 h before the fluorescence measurement to allow the interactions to reach equilibrium.

### 3.4. Preparation of HPLC Columns

The chiral packing materials were prepared by coating Am-**1b** on macroporous silica gel according to a method reported previously [24,25], followed by evaporation of the solvent under reduced pressure. The weight ratio of amylose derivative/silica gel was approximately 1:4, which was estimated by thermogravimetric analysis (TGA). After fractionating with sieves, the obtained silica gel was packed into a stainless steel column (25 cm × 0.20 cm (i.d.)) by a slurry packing technique using a Chemco ECONO-PACKER MODEL CPP-085 (Chemco, Osaka, Japan). The number of theoretical plates per column was estimated to be approximately 3000 for benzene using ethanol as the eluent at a flow rate of 0.05 mL·min^−1^. 1,3,5-Tri-*t*-butylbenzene was used as a non-retained compound to estimate the hold-up time (*t*_0_) [26].

### 3.5. Instruments

NMR spectra were taken on a JNA-LA 400 (JEOL, Tokyo, Japan) (400 MHz for ^1^H) or a JNM-ECA 500 (JEOL) (500 MHz for ^1^H, 125 MHz for ^13^C) spectrometer in CDCl_3_, pyridine-*d*_5_ and DMSO-*d*_6_ using tetramethylsilane or a solvent residual peak as the internal standard. Melting points were measured on a Yanako melting point apparatus and were uncorrected. TGA was conducted with a TG/DTA6200 (SII NanoTechnology, Chiba, Japan) at a heating rate of 10 °C·min^−1^ under a nitrogen flow. IR spectra were obtained using a JASCO (Hachioji, Japan) Fourier Transform IR-460 spectrophotometer with a KBr pellet. Absorption and CD spectra were measured at 25 °C in a 10 mm quartz cell using a JASCO V-570 and a JASCO J-725 spectrometers, respectively. The temperature was controlled using a JASCO ETC-505T (absorption spectroscopy) and a JASCO PTC-348WI apparatus (CD spectroscopy). DLS measurements were performed on a Nano partica SZ-100 (Horiba, Kyoto, Japan) equipped with a 10 mW a diode pumped solid state laser (532 nm) at 25 °C. Fluorescence emission spectra were measured with a JASCO FP-6300. The fluorescence quantum yield was estimated using quinine sulfate in ethanol as a standard material. Chromatographic separations of enantiomers were performed using a JASCO PU-2080 Intelligent HPLC pump equipped with a JASCO MD-2018 and a CD detector (JASCO CD-2095) at ca. 20 °C. A solution of a chiral compound was injected into the chromatographic system by using a Rheodyne Model 7125 injector (Rheodyne, Rohnert Park, CA, USA). Elemental analyses were performed by the Research Institute for Instrumental Analysis of Advanced Science Research Center, Kanazawa University, Kanazawa, Japan.

## 4. Conclusions

We synthesized fluorescent phenylcarbamate derivatives of amylose (Am-**1b**) and β-cyclodextrin (CyD-**1b**) containing terthienyl-based π-conjugated pendant groups. The chiral recognition abilities of Am-**1b** and CyD-**1b** were investigated by measuring their enantioselective fluorescence responses toward chiral aromatic nitro compounds. Am-**1b** and CyD-**1b** exhibited characteristic enantioselectivity for various types of aromatic nitro compounds containing either central or axial chirality. Because both fluorophores consist of the same α-d-glucose-based repeating unit, we can conclude that the chiral recognition abilities of Am-**1b** and CyD-**1b** are mainly derived from their characteristic backbone structures, that is, the one-handed helical structure and cyclic structure, respectively, rather than the chirality of the monomeric units. Although the chiral recognition ability of the present sensors is not adequate, we believe that an appropriate structural design will provide more potent saccharide-based chiral fluorescent sensors. Systematic structural modifications are currently in progress and will be reported in due course.

## Figures and Tables

**Table 1:** Resolutions of racemates on Am-**1**- and ATPC-based CSPs.

Racemates	Am-1		ATPC	
	*k*_1_	α	*k*_1_	α
**11a**	1.15 (*R*)	1.17	1.40 (*R*)	ca. 1
**11b**	0.29	1.0	0.95 (*R*)	1.22
**11c**	0.14	1.0	0.39	1.0

Column: 25 cm × 0.20 cm (i.d.). Eluent: ethanol. Flow rate: 0.05 mL·min^−1^. The signs in parentheses represent the absolute configurations of the first-eluted enantiomers.

## Supplementary Materials

Supplementary materials can be accessed at: http://www.mdpi.com/1420-3049/21/11/1518/s1.
