# Supplementary material for: Development of Amylose- and β-Cyclodextrin-Based Chiral Fluorescent Sensors Bearing Terthienyl Pendants
Source: Molecules. 2016 Nov 11;21(11):1518. doi: 10.3390/molecules21111518 (PMC6274270; doi:10.3390/molecules21111518)
Supplement: Supplementary file 1 [file molecules-21-01518-s001.pdf]

Supplementary Materials: Development of Amylose- and β-Cyclodextrin-Based Chiral Fluorescent Sensors Bearing Terthienyl Pendants

Tomoyuki Ikai, Changsik Yun, Yutaka Kojima, Daisuke Suzuki, Katsuhiro Maeda and Shigeyoshi Kanoh

1. Supporting Data

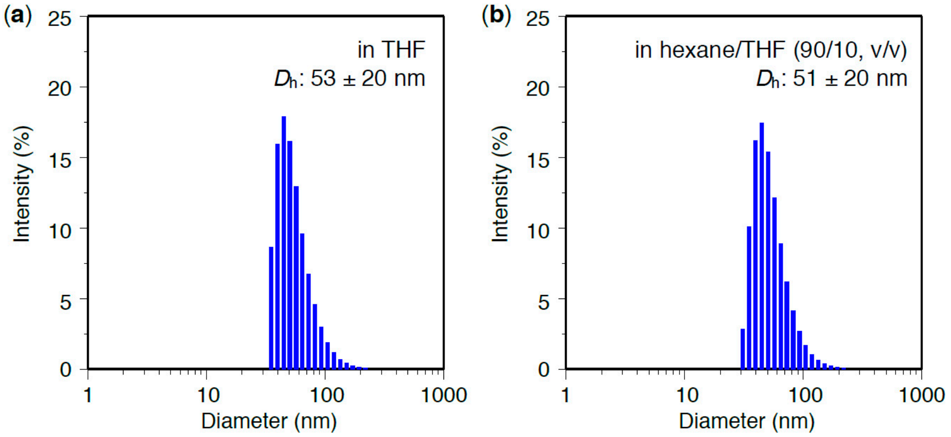

Figure S1. Histogram analysis of the DLS measurements of Am-**1b** in THF (**a**) and hexane/THF (90/10, *v*/*v*) (**b**) at 30 °C after filtration through a membrane filter with a pore size of 0.45 µm. [Glucose unit] = 1.0×10^{-5} M.

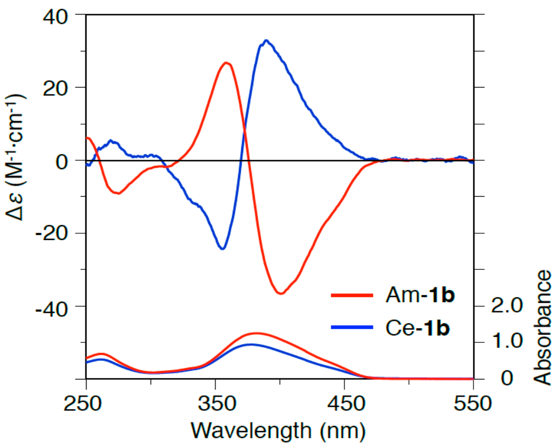

Figure S2. CD and absorption spectra of Am-**1b** and Ce-**1b** in hexane/THF (90/10, *v*/*v*) at 25 °C. [Glucose unit] = 1.0×10^{-5} M. (Reproduced with permission from Reference [1]. Copyright 2016 The Royal Society of Chemistry).

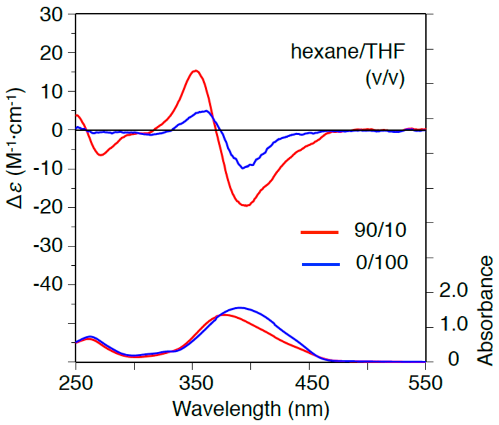

Figure S3. CD and absorption spectra of CyD-**1b** in THF (blue line) and hexane/THF (90/10, *v*/*v*) (red line) at 25 °C. [Glucose unit] = 1.0 × 10^{-5} M.

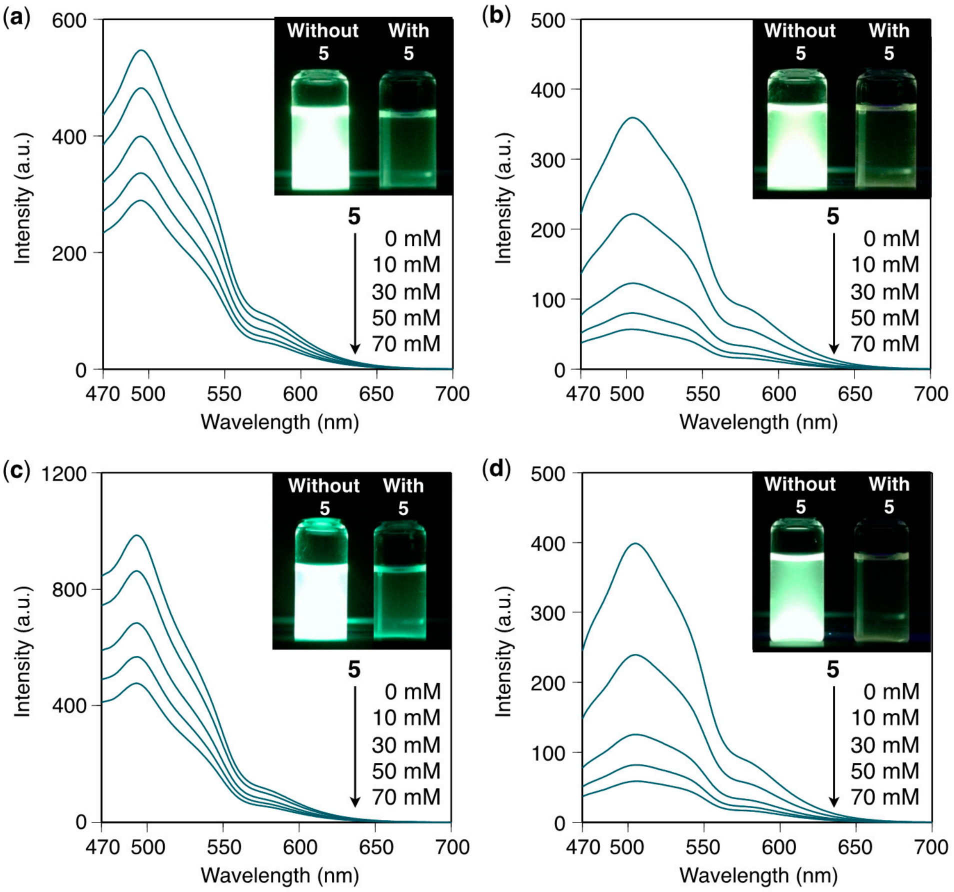

Figure S4. Fluorescence spectra of Am-**1b** (**a**,**b**) and CyD-**1b** (**c**,**d**) upon the addition of various amounts of **5** (0–70 mM) in THF (**a**,**c**) and hexane/THF (90/10, *v*/*v*) (**b**,**d**) at room temperature. [Glucose unit] = 1.0 × 10^{-5} M, λ_{ex} = 450 nm. Insets: Photographs of the corresponding solutions in the absence (left) and presence (right) of nitrobenzene (70 mM) under irradiation at 365 nm.

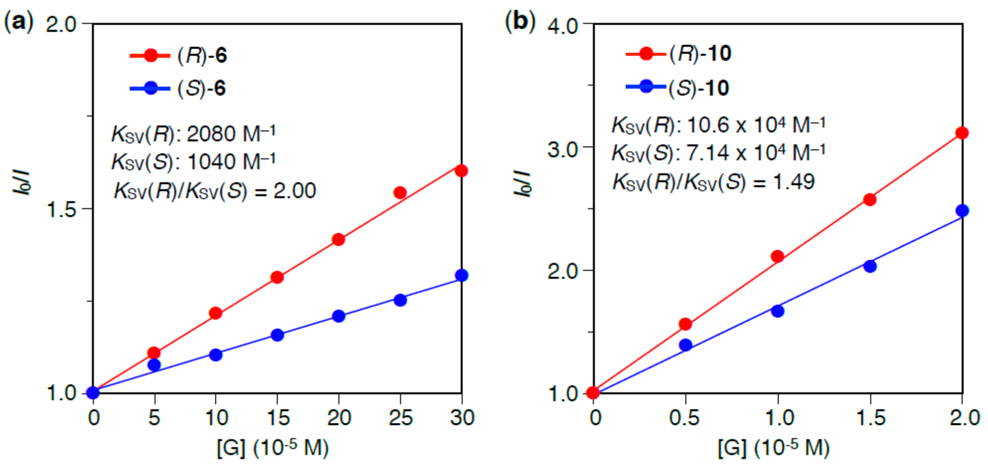

Figure S5. Stern–Volmer plots for the fluorescence quenching of Ce-**1b** (λ_{ex} = 450 nm) by the (*R*)- (red) and (*S*)- (blue) isomers of **6** (**a**) and **10** (**b**) in hexane/THF (90/10, *v*/*v*) at room temperature. [Glucose unit] = 1.0 × 10^{-5} M. The results of the fluorescence quenching using **7a**, **8**, **9** and **11a** as guest quenchers were reported in Reference [1].

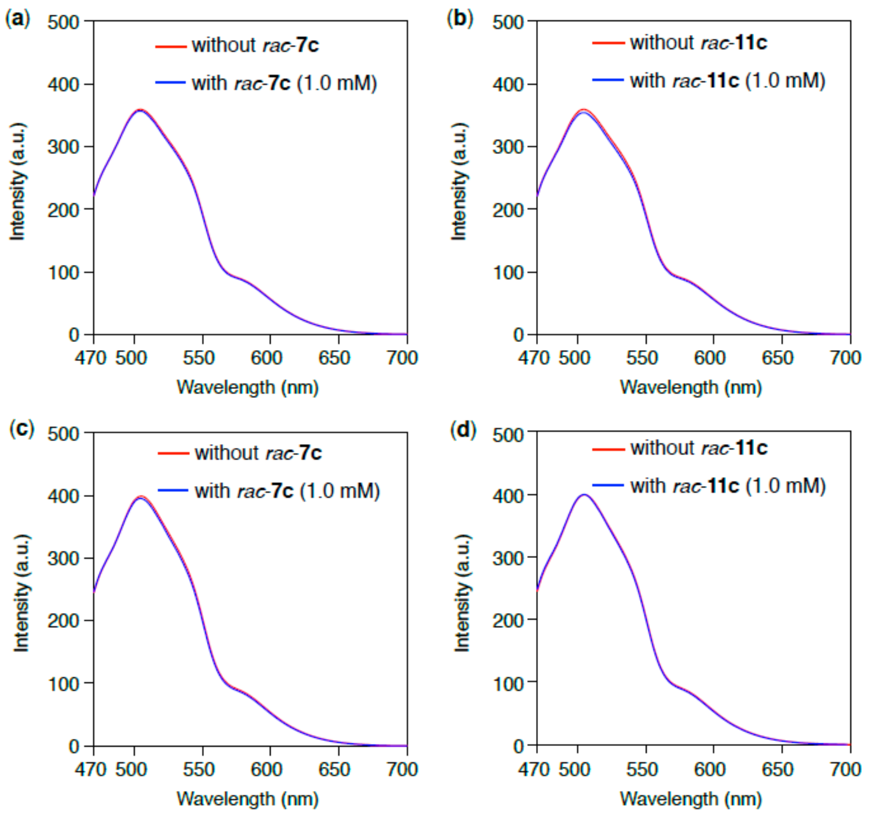

Figure S6. Fluorescence spectra of Am-**1b** (**a**,**b**) and CyD-**1b** (**c**,**d**) in the absence (red line) and presence (blue line) of *rac*-**7c** (**a**,**c**) and *rac*-**11c** (**b**,**d**) in hexane/THF (90/10, *v*/*v*) at room temperature. [Glucose unit] = 1.0 × 10^{-5} M, λ_{ex} = 450 nm.

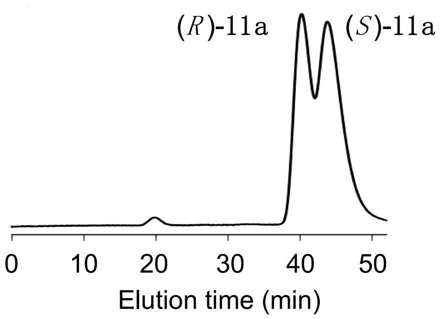

Figure S7. Chromatogram for the resolution of **11a** on Am-**1b**-based CSP (column: 25 cm × 0.20 cm (i.d.); eluent: ethanol; flow rate: 0.05 mL·min^{-1}).

2. NMR Spectral Data

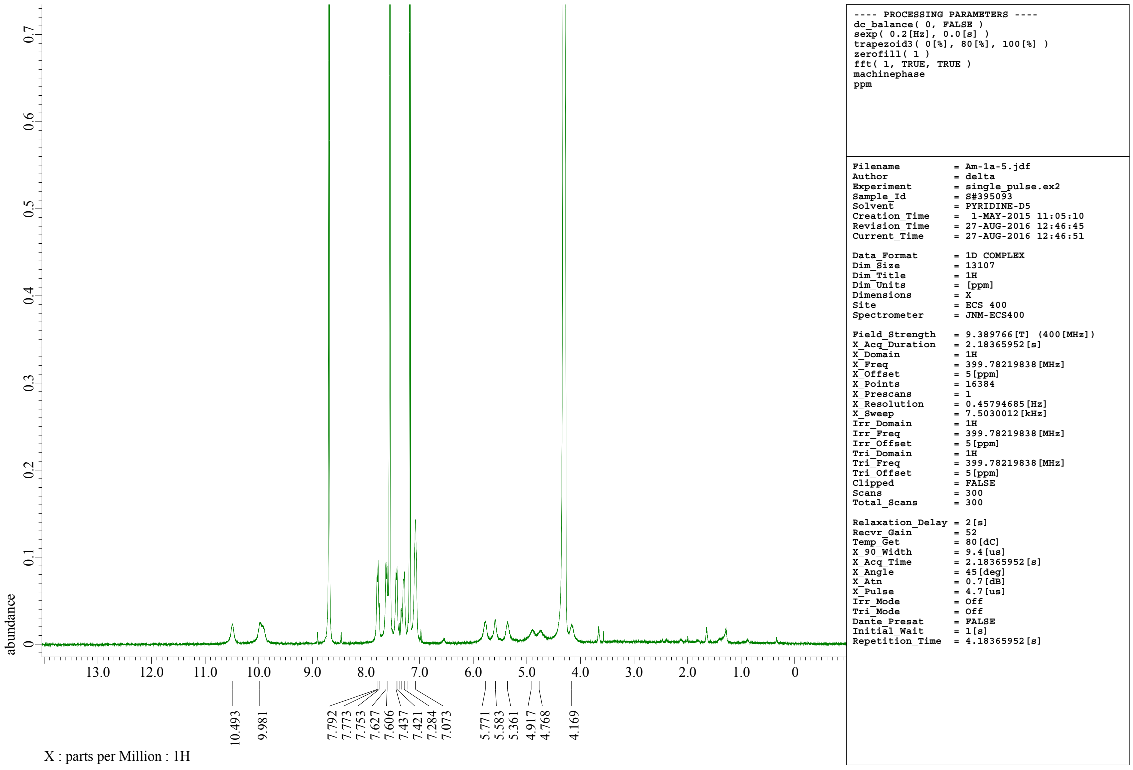

Figure S8. ^{1}H-NMR (pyridine-*d*5, 400 MHz, 80 °C) spectrum of Am-**1a**.

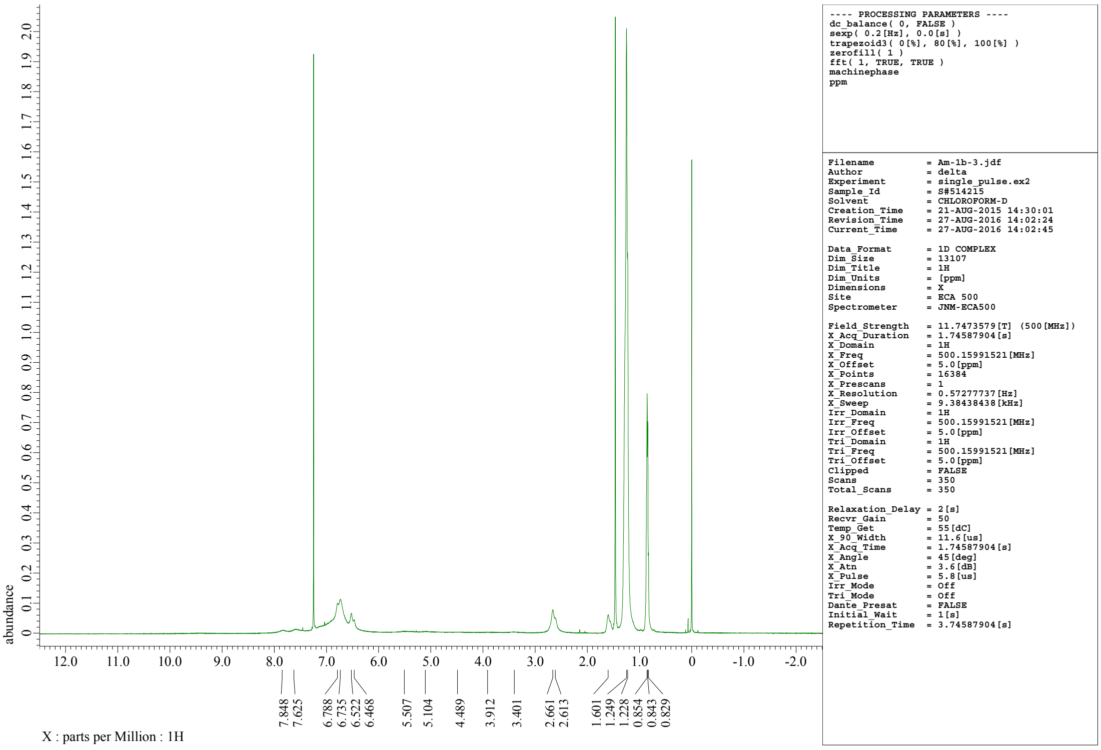

Figure S9. ^{1}H-NMR ($CDCl_3$, 500 MHz, 55 °C) spectrum of Am-**1b**.

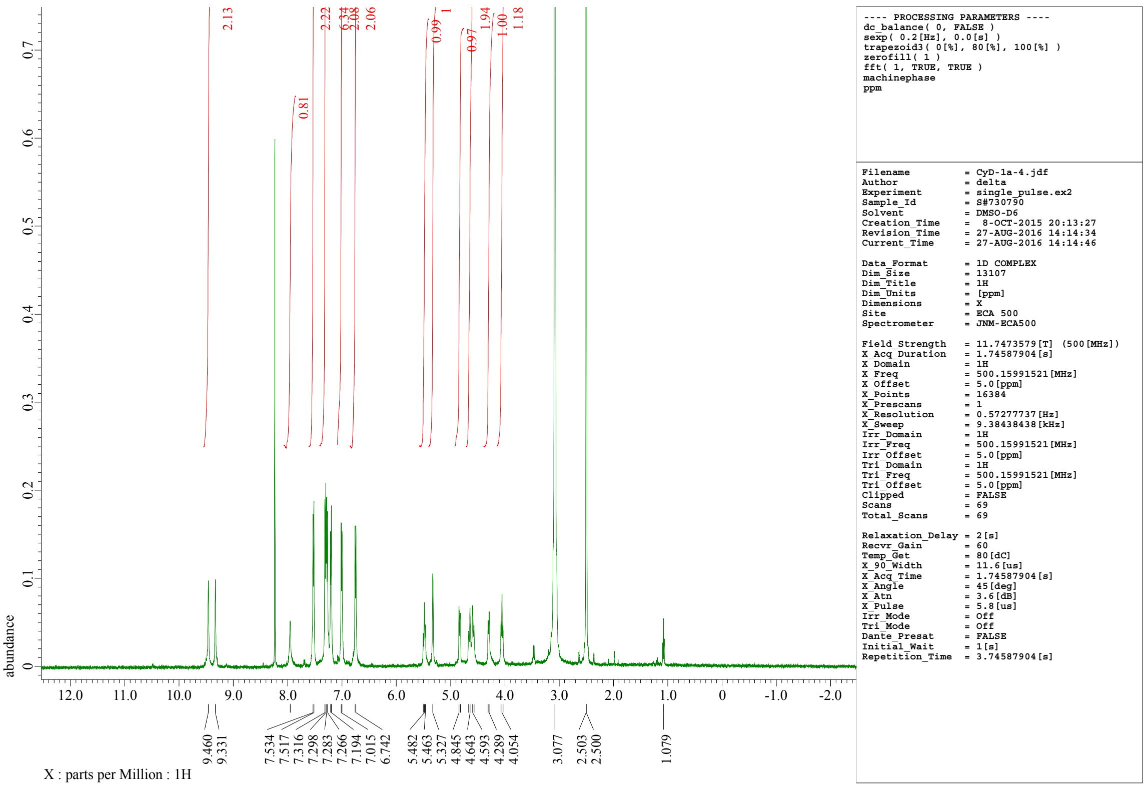

Figure S10. ^{1}H-NMR (DMSO-d_6, 500 MHz, 80 °C) spectrum of CyD-**1a**.

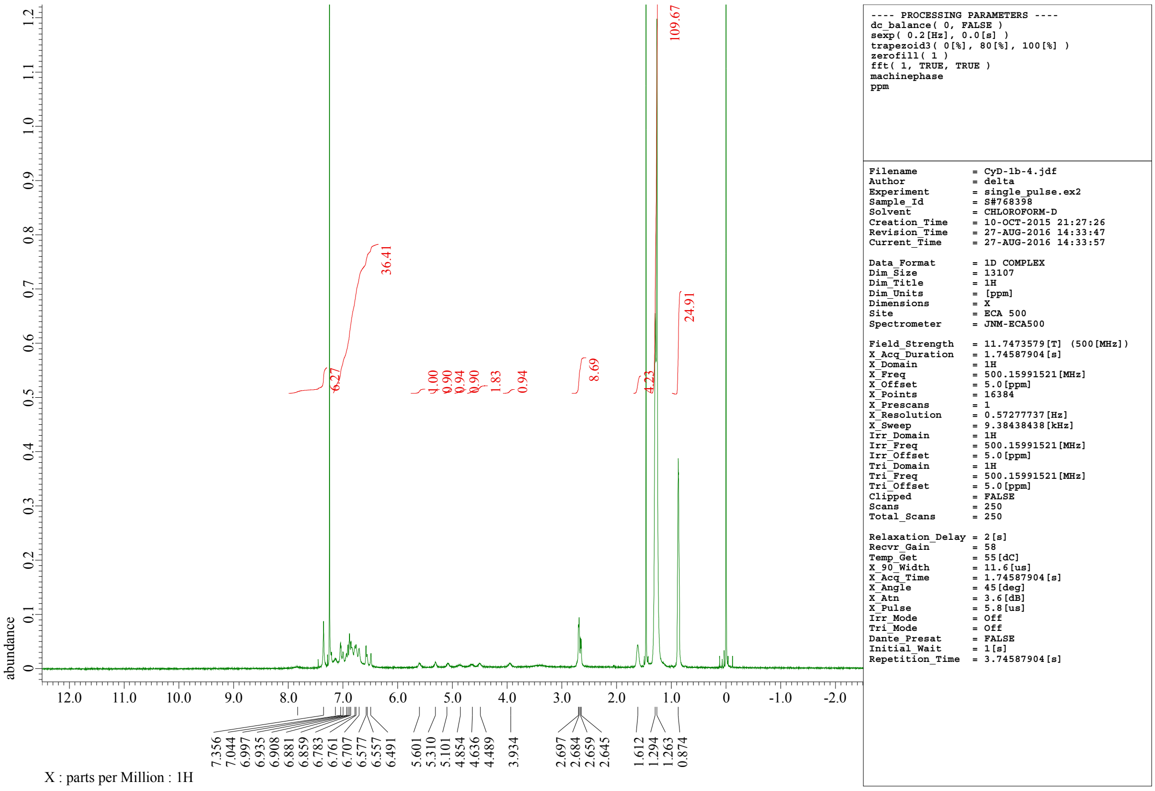

Figure S11. ^{1}H-NMR ($CDCl_3$, 500 MHz, 55 °C) spectrum of CyD-**1b**.

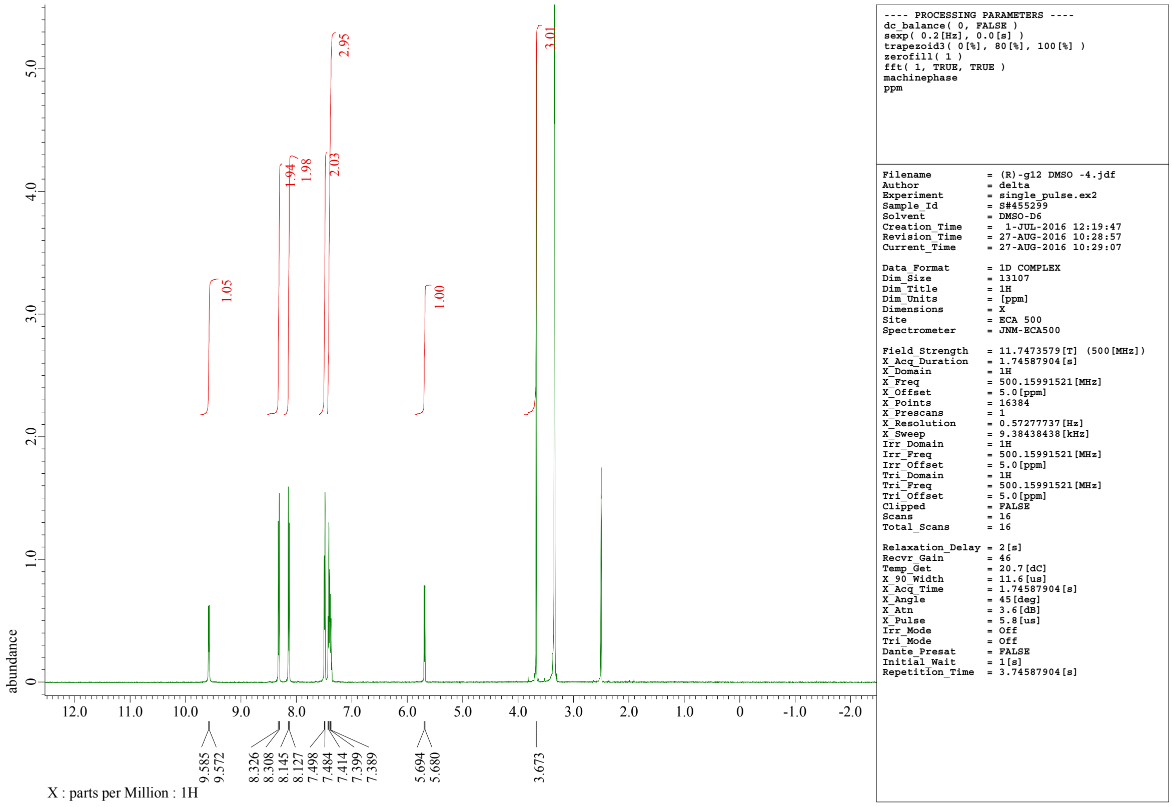

Figure S12. ^{1}H-NMR (DMSO-d_6, 500 MHz, rt) spectrum of (*R*)-**6**.

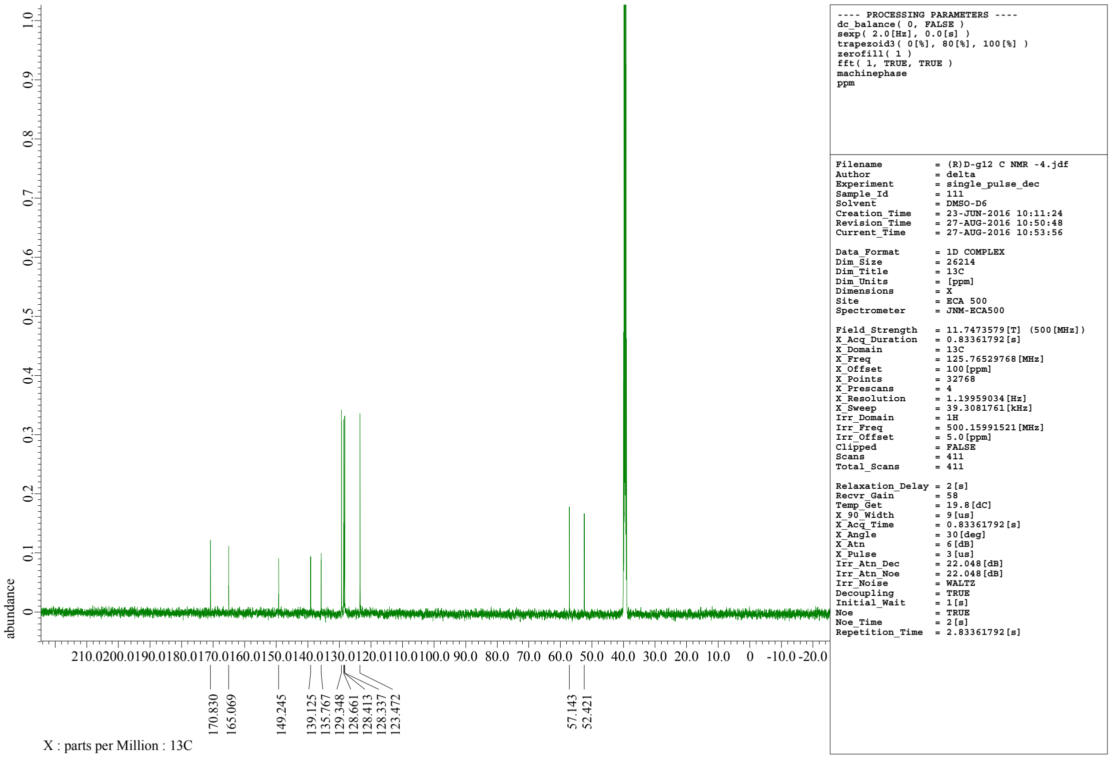

Figure S13. ^{13}C-NMR (DMSO-d_6, 125 MHz, rt) spectrum of (*R*)-**6**.

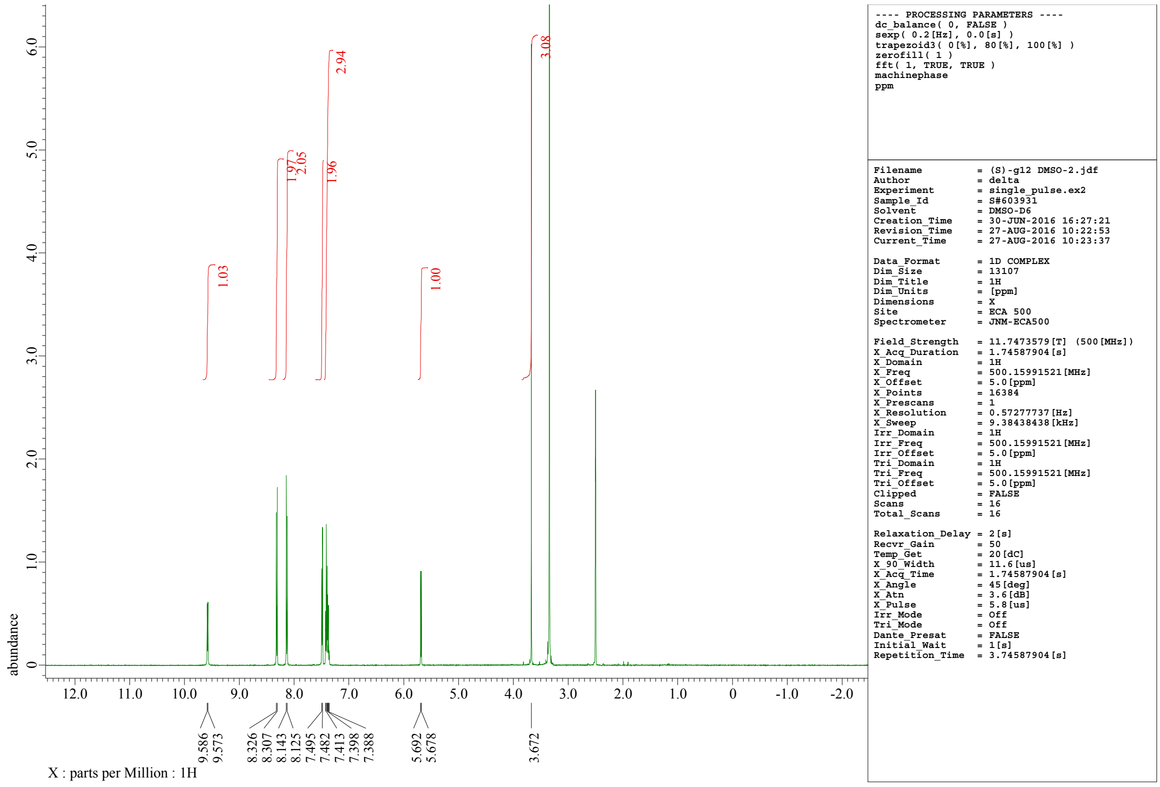

Figure S14. ^{1}H-NMR (DMSO-d_6, 500 MHz, rt) spectrum of (*S*)-**6**.

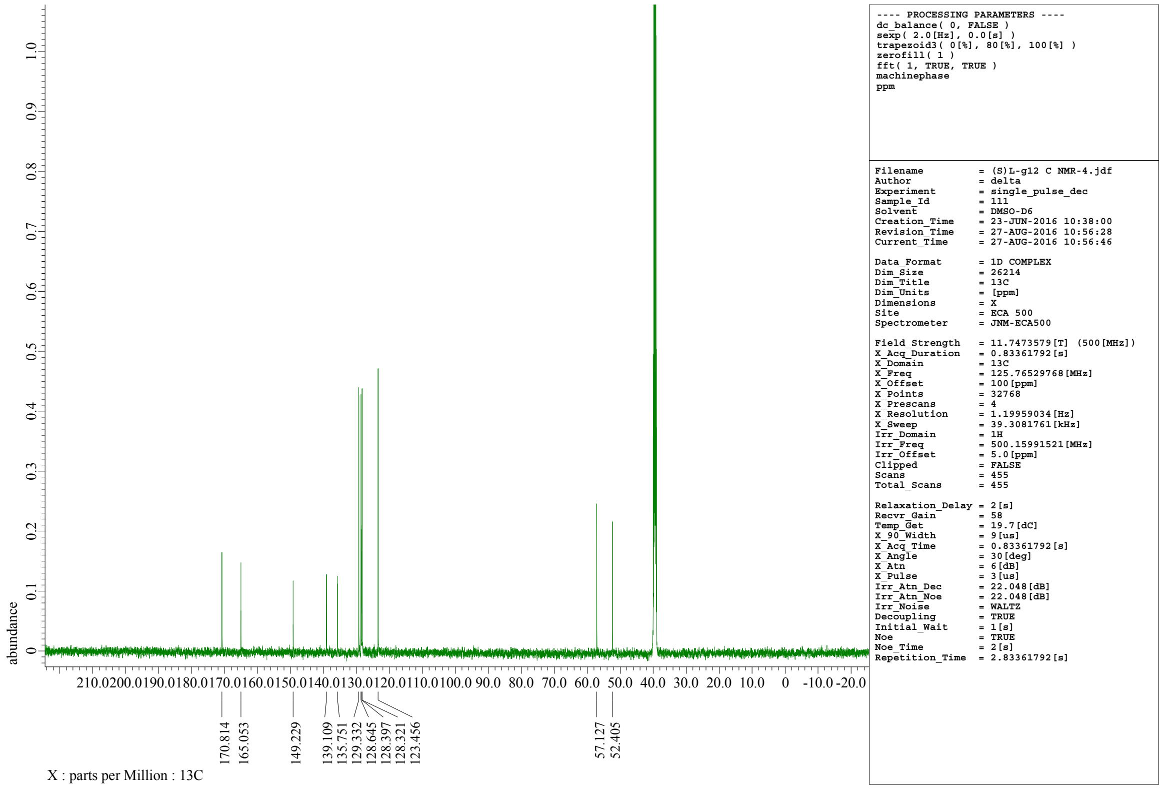

Figure S15. ^{13}C-NMR (DMSO-d_6, 125 MHz, rt) spectrum of (*S*)-**6**.

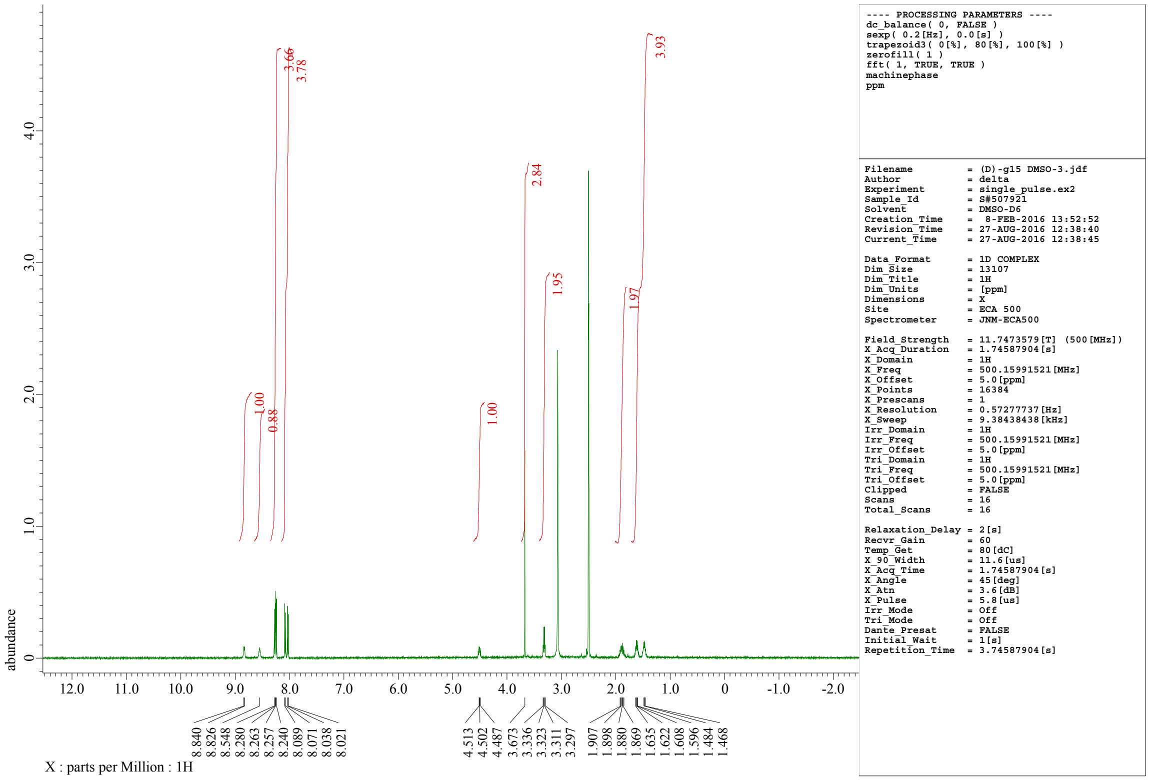

Figure S16. ^{1}H-NMR (DMSO-d_6, 500 MHz, 80 °C) spectrum of (*R*)-**10**.

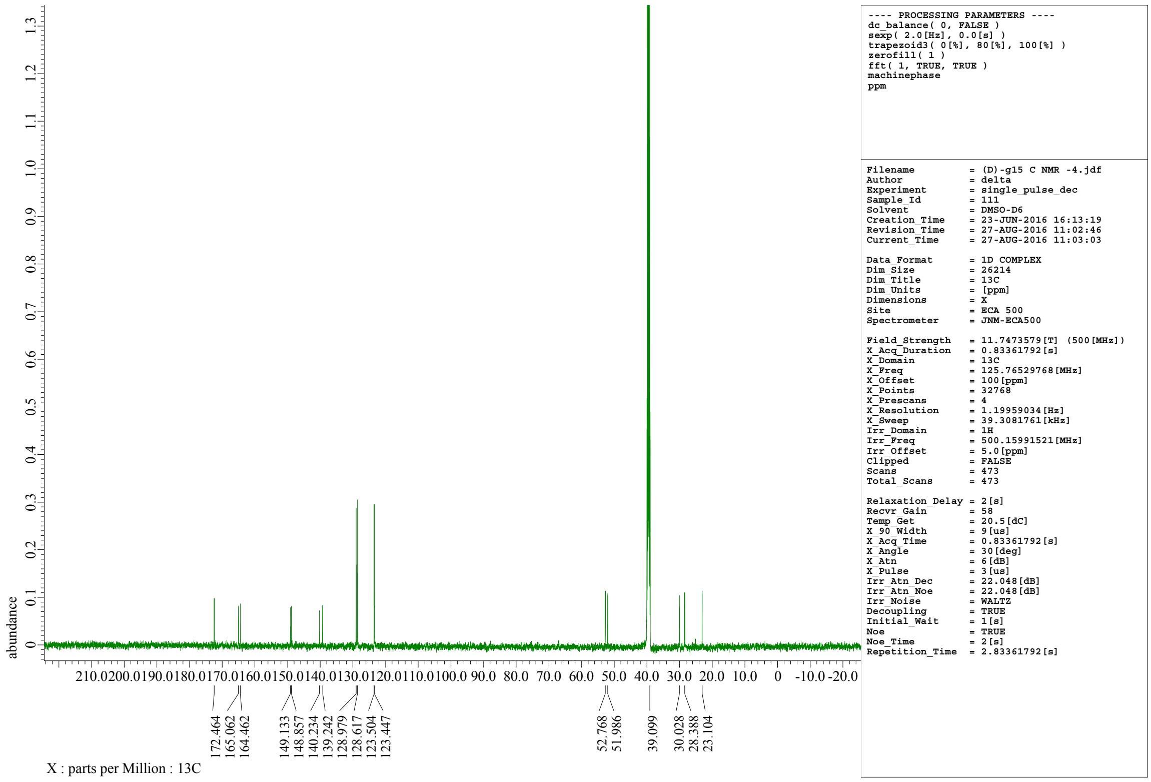

Figure S17. ^{13}C-NMR (DMSO-d_6, 125 MHz, rt) spectrum of (*R*)-**10**.

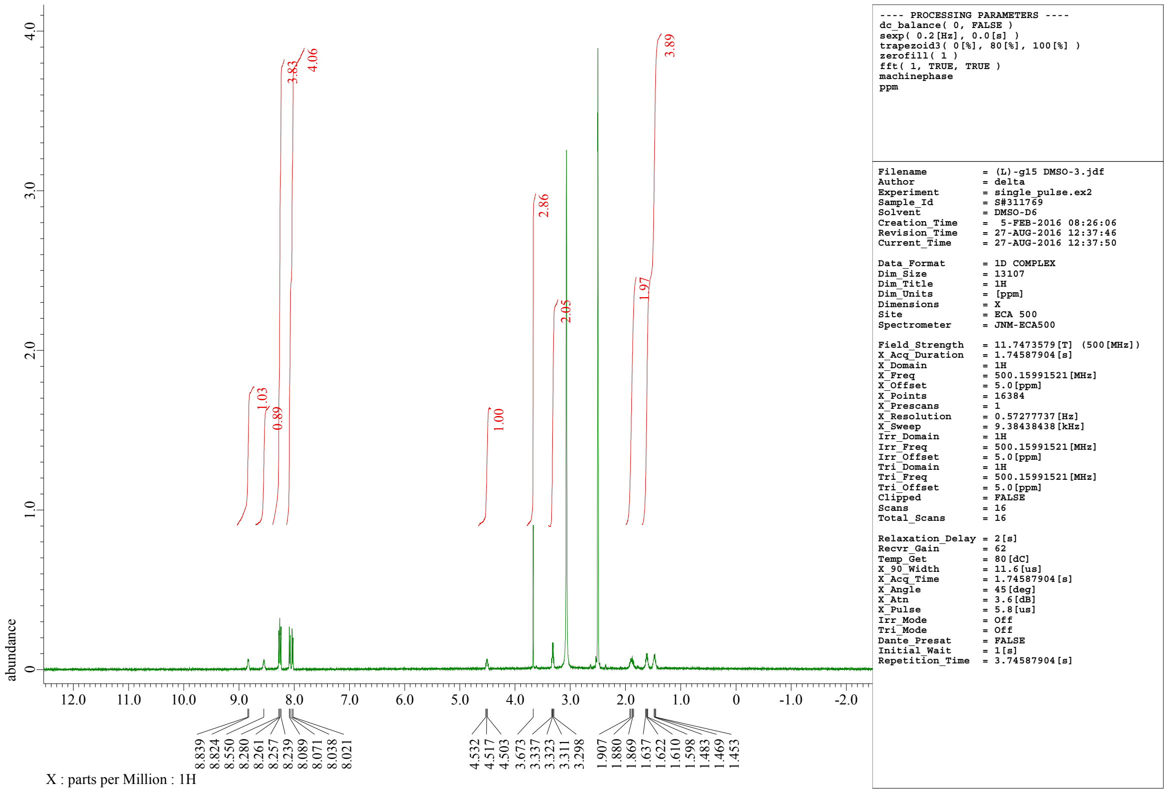

Figure S18. ^{1}H-NMR (DMSO-*d*$_6$, 500 MHz, 80 °C) spectrum of (*S*)-**10**.

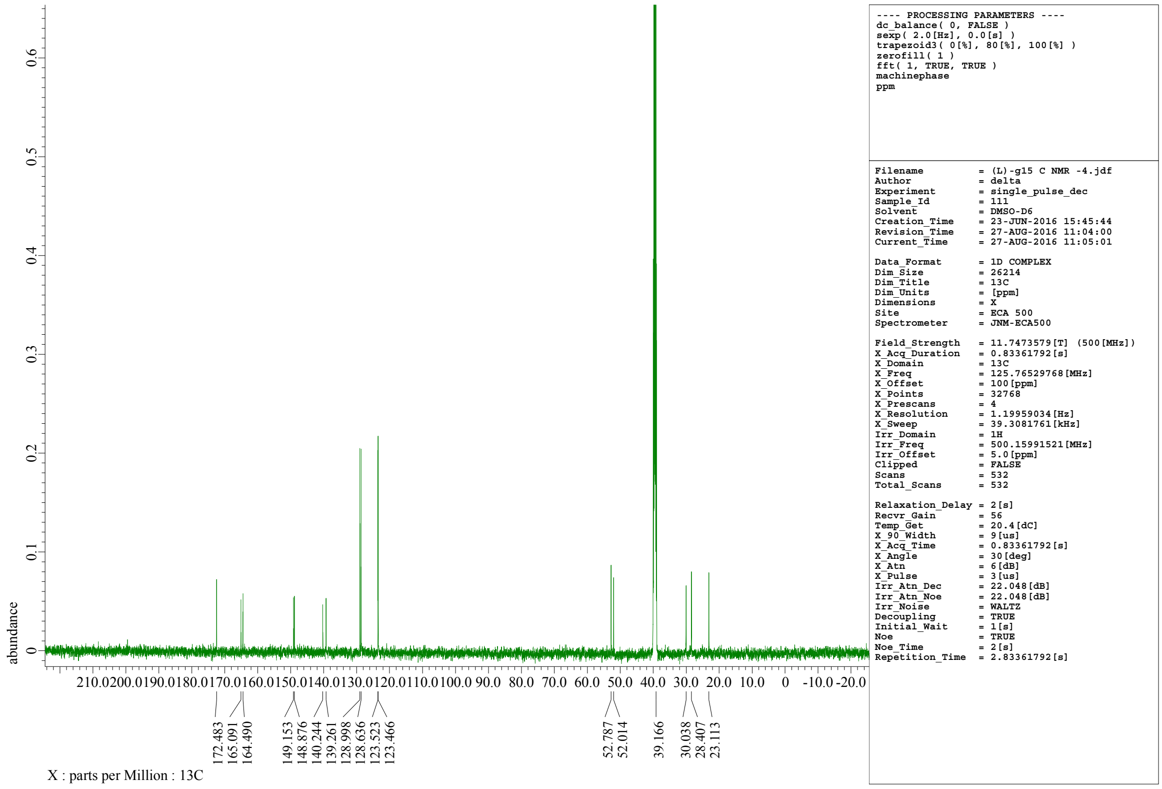

Figure S19. ^{13}C-NMR (DMSO-*d*$_6$, 125 MHz, rt) spectrum of (*S*)-**10**.

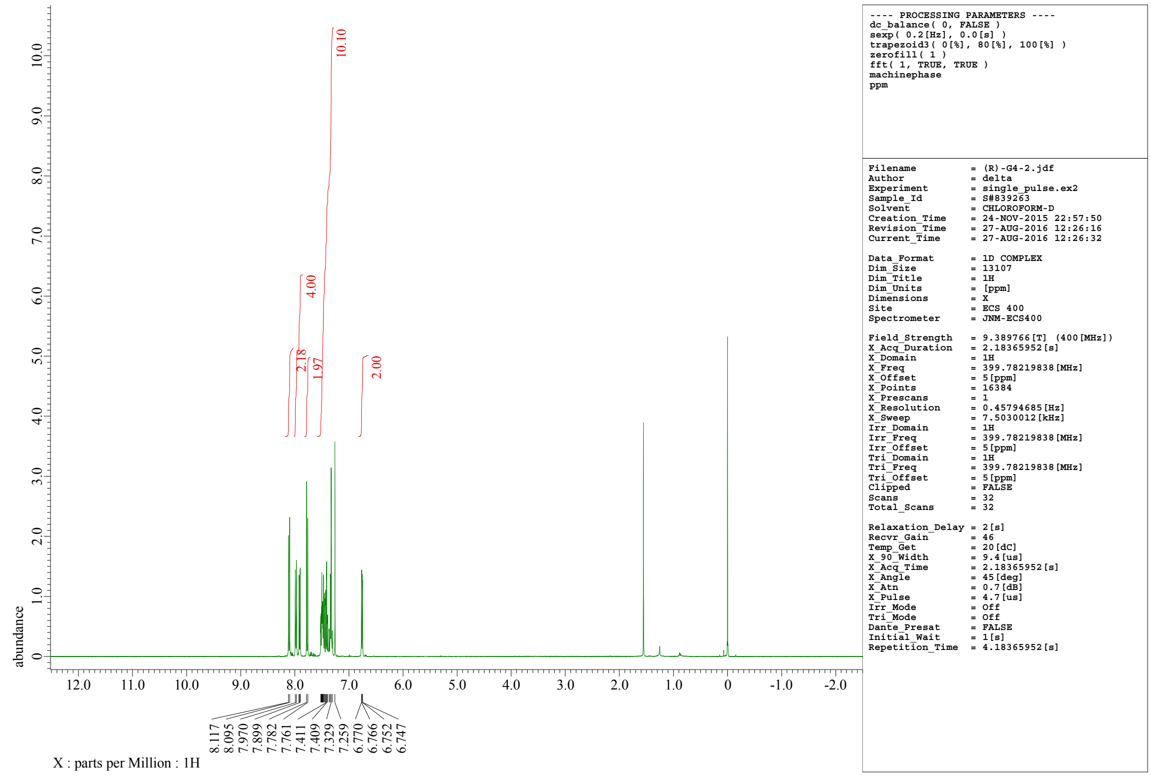

Figure S20. ^{1}H-NMR ($CDCl_3$, 400 MHz, rt) spectrum of (*R*)-**11c**.

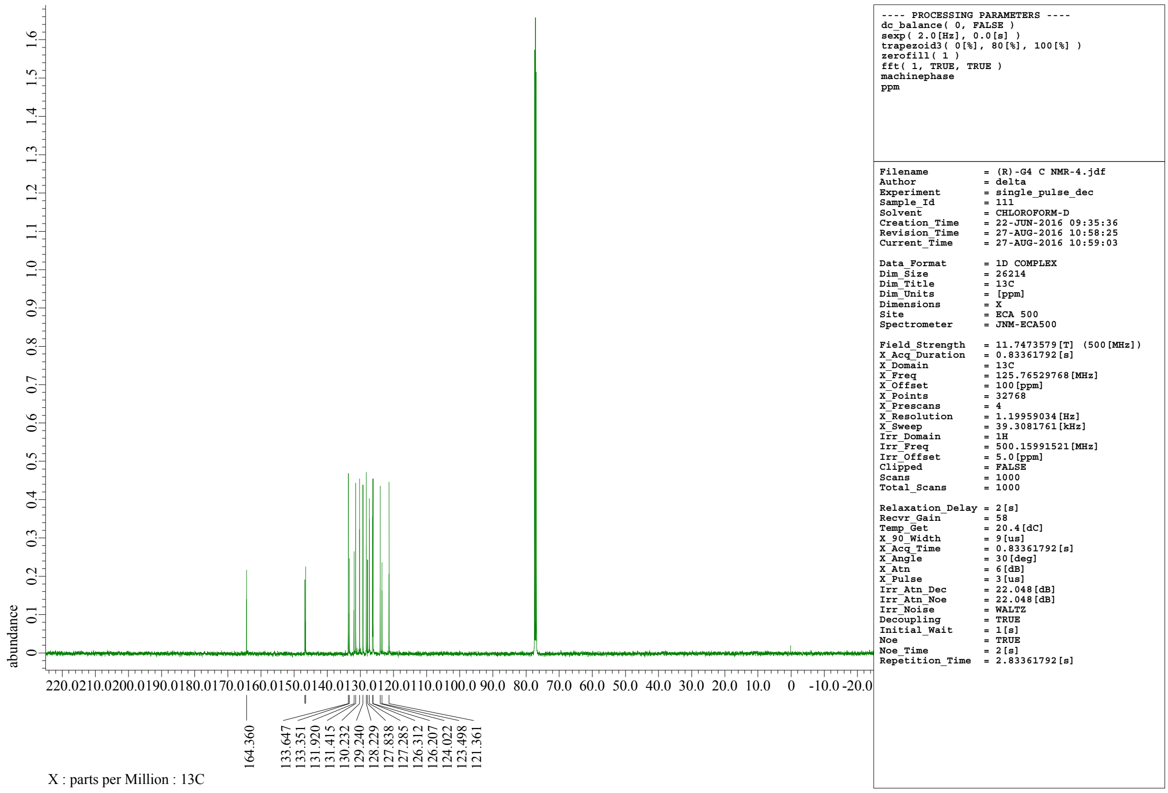

Figure S21. ^{13}C-NMR ($CDCl_3$, 125 MHz, rt) spectrum of (*R*)-**11c**.

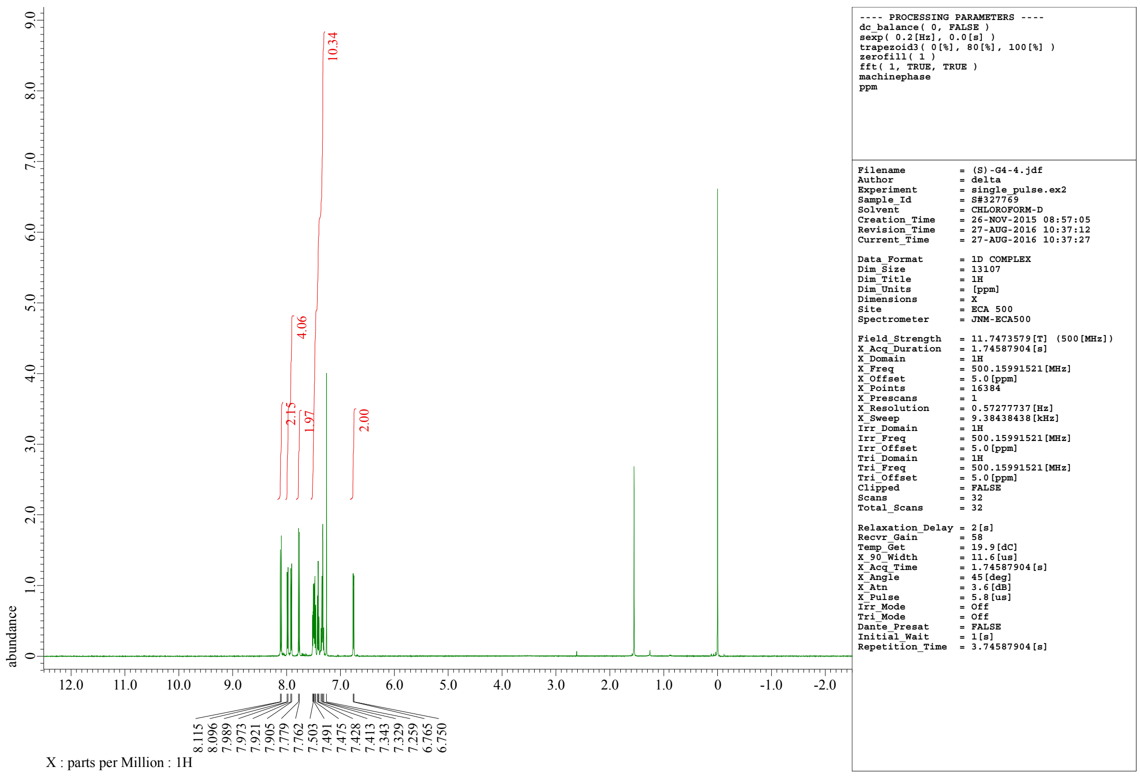

Figure S22. ^{1}H-NMR ($CDCl_3$, 500 MHz, rt) spectrum of (*S*)-**11c**.

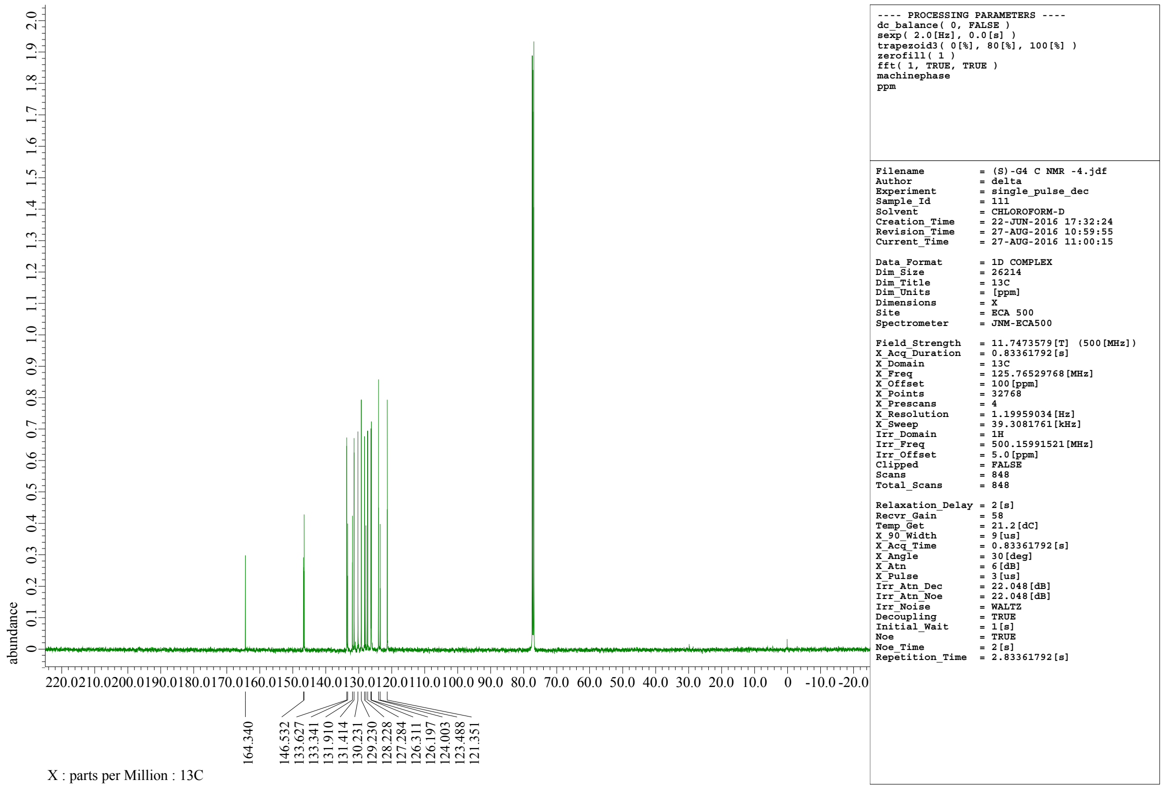

Figure S23. ^{13}C-NMR ($CDCl_3$, 125 MHz, rt) spectrum of (*S*)-**11c**.

Reference

1. Ikai, T.; Suzuki, D.; Kojima, Y.; Yun, C.; Maeda, K.; Kanoh, S. Chiral fluorescent sensors based on cellulose derivatives bearing terthienyl pendants. *Polym. Chem.* **2016**, *7*, 4793–4801.
